# Genomic insights into novel extremotolerant bacteria isolated from the NASA Phoenix mission spacecraft assembly cleanrooms

**DOI:** 10.1186/s40168-025-02082-1

**Published:** 2025-05-12

**Authors:** Júnia Schultz, Tahira Jamil, Pratyay Sengupta, Shobhan Karthick Muthamilselvi Sivabalan, Anamika Rawat, Niketan Patel, Srinivasan Krishnamurthi, Intikhab Alam, Nitin K. Singh, Karthik Raman, Alexandre Soares Rosado, Kasthuri Venkateswaran

**Affiliations:** 1 Biological and Environmental Science and Engineering Division, King Abdullah University of Science and Technology, Makkah, Thuwal 23955 Saudi Arabia; 2 Department of Biotechnology, Bhupat and Jyoti Mehta School of Biosciences, Indian Institute of Technology Madras, Chennai, Tamil Nadu 600036 India; 3 Center for Integrative Biology and Systems Medicine (IBSE), Indian Institute of Technology Madras, Chennai, Tamil Nadu 600036 India; 4 Robert Bosch Centre for Data Science and Artificial Intelligence (RBCDSAI), Indian Institute of Technology Madras, Chennai, 600036 Tamil Nadu India; 5 Microbial Type Culture Collection and Gene Bank (MTCC), Institute of Microbial Technology, Chandigarh, 160036 India; 6 NASA Jet Propulsion Laboratory, California Institute of Technology, Pasadena, CA USA; 7 Department of Data Science and AI, Wadhwani School of Data Science and AI, Indian Institute of Technology Madras, Chennai, Tamil Nadu 600036 India; 8 Bioscience Program, Biological and Environmental Science and Engineering (BESE), Division, King Abdullah University of Science and Technology (KAUST), Makkah, Thuwal 23955 Saudi Arabia

**Keywords:** Extreme environment, Cleanroom, Phylogenomics, Novel species, Phoenix mission, BGCs

## Abstract

**Background:**

Human-designed oligotrophic environments, such as cleanrooms, harbor unique microbial communities shaped by selective pressures like temperature, humidity, nutrient availability, cleaning reagents, and radiation. Maintaining the biological cleanliness of NASA’s mission-associated cleanrooms, where spacecraft are assembled and tested, is critical for planetary protection. Even with stringent controls such as regulated airflow, temperature management, and rigorous cleaning, resilient microorganisms can persist in these environments, posing potential risks for space missions.

**Results:**

During the Phoenix spacecraft mission, genomes of 215 bacterial isolates were sequenced and based on overall genome-related indices, 53 strains belonging to 26 novel species were recognized. Metagenome mapping indicated less than 0.1% of the reads associated with novel species, suggesting their rarity. Genes responsible for biofilm formation, such as BolA (COG0271) and CvpA (COG1286), were predominantly found in proteobacterial members but were absent in other non-spore-forming and spore-forming species. YqgA (COG1811) was detected in most spore-forming members but was absent in *Paenibacillus* and non-spore-forming species. Cell fate regulators, COG1774 (YaaT), COG3679 (YlbF, YheA/YmcA), and COG4550 (YmcA, YheA/YmcA), controlling sporulation, competence, and biofilm development processes, were observed in all spore-formers but were missing in non-spore-forming species. COG analyses further revealed resistance-conferring proteins in all spore-formers (*n* = 13 species) and eight actinobacterial species, responsible for enhanced membrane transport and signaling under radiation (COG3253), transcription regulation under radiation stress (COG1108), and DNA repair and stress responses (COG2318). Additional functional analysis revealed that *Agrococcus phoenicis*, *Microbacterium canaveralium*, and *Microbacterium* jpeli contained biosynthetic gene clusters (BGCs) for ε-poly-L-lysine, beneficial in food preservation and biomedical applications. Two novel *Sphingomonas* species exhibited for zeaxanthin, an antioxidant beneficial for eye health. *Paenibacillus canaveralius* harbored genes for bacillibactin, crucial for iron acquisition. *Georgenia phoenicis* had BGCs for alkylresorcinols, compounds with antimicrobial and anticancer properties used in food preservation and pharmaceuticals.

**Conclusion:**

Despite stringent decontamination and controlled environmental conditions, cleanrooms harbor unique bacterial species that form biofilms, resist various stressors, and produce valuable biotechnological compounds. The reduced microbial competition in these environments enhances the discovery of novel microbial diversity, contributing to the mitigation of microbial contamination and fostering biotechnological innovation.

Video Abstract

**Supplementary Information:**

The online version contains supplementary material available at 10.1186/s40168-025-02082-1.

## Introduction

Cleanrooms and other human-designed oligotrophic environments present distinct ecosystems that may expedite microbial speciation due to unique selective pressures [1]. These pressures may arise from specialized construction materials, controlled temperature and humidity, and exposure to cleaning agents, diverging from more nutrient-rich natural settings [2]. Such environments select microbes that can survive nutrient-poor conditions, potentially giving rise to new species [3]. Globally, human activities transport microbes to different oligotrophic environments, like cleanrooms, facilitating distinctive evolutionary trajectories [2]. Despite the resource-limited conditions, microbial communities in these controlled environments are complex and competitive. The isolation of rare microbes from cleanroom environments is influenced by long-term selective pressures, such as desiccation, repeated sterilization, and low-nutrient availability, which shape microbial survival strategies over years to decades [4, 5].

Ensuring the biological cleanliness of the National Aeronautics and Space Administration’s (NASA) mission-associated cleanrooms, where spacecraft are assembled and tested, is imperative to meet planetary protection requirements [6]. These facilities undergo constant monitoring to detect and assess the presence of any microorganisms that could potentially survive a transfer to an extraterrestrial environment via robotic exploration devices [7–9]. Despite meticulous control measures, including regulation of airflow, humidity, temperature, and air particulate concentrations, along with rigorous cleaning using chemical detergents, UV radiation, and hydrogen peroxide, certain microorganisms can persist in this challenging and nutrient-limited environment [5, 10–12].

The “cleanroom effect” may provide a platform for microorganisms to adapt to selective pressures (i.e., extreme oligotrophy, low-humidity, and desiccation conditions), bolstering their growth, survival, lifestyle, and resilience under extreme conditions, and the production of specialized metabolites [12, 13]. It is crucial to characterize these resistant microbes, which defy conventional biological control measures and potentially identify novel microbial species. This effort is pivotal for monitoring the risk of forward microbial contamination and safeguarding extraterrestrial environments against unintentional colonization of exploring planets [14].

During the Phoenix mission, 215 strains were isolated from the Kennedy Space Center-Payload Hazardous Servicing Facility (KSC-PHSF) cleanroom floors under various extreme conditions [10], and whole genome sequencing (WGS) of all 215 isolates was performed during this study. The central objectives of this study were to characterize a cohort of 53 strains, representing 26 previously unidentified bacterial species discovered among Phoenix mission isolates. These strains were subjected to extensive examination, which included characterizing their physiological attributes, and conducting thorough genome analysis, followed by in-depth phylogenomic assessments. Evaluations were performed to determine the incidence, prevalence, and persistence of these novel species even after 9 years by analyzing metagenomic reads sourced from several NASA cleanrooms, including KSC-PHSF. In parallel, an investigation into the genomic functions of these extremotolerants was undertaken, with a particular emphasis on the discovery of potential genes responsible for radiation resistance and secondary metabolites, indicative of their adaptive capacity and biotechnological applications (Supplementary Figure S1).

## Results

Based on WGS, the bacterial strains (*n* = 215) isolated from the KSC-PHSF were classified into three phyla: *Actinomycetota*, *Bacillota*, and *Pseudomonadota*. Furthermore, around 25% of the bacterial strains (53 out of 215 isolates) were identified as belonging to 26 novel species and most of them belong to the members of the class *Bacilli* (47.7%), *Alphaproteobacteria* (24.5%), *Gammaproteobacteria* (13.9%), and *Actinomycetia* (13.9%). The percent occurrence of the novel species at the family level is given in Supplementary Figure S2. Among 53 strains, these 26 species had not been previously described, encompassing 18 genera. Within these 53 novel bacterial strains, 33 strains were isolated before the arrival of the Phoenix mission spacecraft to the KSC-PHSF cleanroom (21 novel species), 7 were cultured during the assembly and testing of the spacecraft (3 novel species), and 13 were isolated from the cleanroom floors after moving the spacecraft for the launch (2 novel species). Among the 53 novel extremo tolerant strains, 22 were isolated under the alkaline condition (> pH 10; alkalotolerant), eight after heat-shock (80 °C; 15 min; heat-tolerant), seven grown at 4 °C (psychrotolerant), six at 25 °C (mesophile), five under anaerobic atmosphere, and five after exposing to UVC condition (254 nm; 1000 J/m^2^). All these cultivation conditions were already published [9].

### Genome features and relatedness indices

The isolation source, conditions, and WGS assembly statistics of the 53 novel strains are presented in Supplementary Table S1. The draft genomes of the novel species generated using the Nanopore platform were constructed with high-quality sequences, with assembly quality ranging from the complete genome (*n* = 20) to 8 scaffolds, and many of the strains exhibited > 99% completeness. The similarities among the closely related species of the novel species based on marker genes (16S rRNA and gyrB), average nucleotide index (ANI), average amino acid index (AAI), and digital DNA:DNA Hybridization (dDDH) are given in Table 1. Moreover, ANI indices (< 95%) and dDDH values (< 70%) fell below the threshold levels of bacterial species identity, confirming that the examined Phoenix mission strains (*n* = 53) were novel species. The ANI index ranged from 79 to 94%, with most of the 53 novel strains having less than 90% of ANI similarity with the closest relatives. Since no set threshold values for AAI and bacterial genus discrimination exist, it could not be definitively determined whether any of these novel species belong to new genera.

**Table 1:** Whole genome, marker genes sequence similarities, and dDDH values between novel bacterial species and nearest neighbor from the Phoenix spacecraft mission

Phoenix novel species	Phoenix strain #	GenBank accession # of 16S rRNA gene of Phoenix isolates	16S rRNA gene characteristics of the closest type strain of the recognized species			GenBank accession # of WGS of Phoenix strains	WGS characteristics of the closest type strain of the recognized species					
			Name	Accession #	Percent similarities		Name	Accession #	ANI (%)	AAI (%)	dDDH (%)	gyrB (%)
*Shouchella phoenicis*	1P01AA^T^	EU977642	*Alkalihalobacillus miscanthi*	NR_180786.1	99.1	JBDFMQ000000000	*Shouchella hunanensis*	GCA_028735875.1	80.5	79.9	20.4	82.8
*Georgenia phoenicis*	1P01AC^T^	EU977812	*Georgenia satyanarayanai*	NR_117051.1	99.3	CP154855	*"Oceanitalea stevensii"*	GCF_014837105.1	90.0	88.2	35.8	N/A
*Lysinibacillus phoenicis*	1P01SD^T^	PP475405	*Lysinibacillus fusiformis*	NR_042072.1	98.6	JBDFMP000000000	*Lysinibacillus capsici*	GCF_020217405.1	87.0	90.0	32.3	91.0
*Agrococcus phoenicis*	1P02AA^T^	PP475406	*Agrococcus terreus*	NR_116650.1	98.6	CP154854	*Agrococcus carbonis*	GCF_900104705.1	86.0	80.0	26.7	90.5
*Alkalihalobacillus phoenicis*	1P02AB^T^	EU977645	*Alkalihalobacillus alcalophilus*	NR_036889.1	99.5	CP154853	*Alkalihalobacillus alcalophilus*	GCF_004802515.1	92.0	93.4	45.1	96.3
*Pseudomonas phoenicis*	1P02AnB^T^	PP475407	*Pseudomonas juntendi*	NR_180497.1	99.0	CP154852	*Pseudomonas cremoricolorata*	GCF_000425745.1	85.6	85.1	27.1	87.2
*Bacillus jepli*	1P02SD^T^	EU977769	*Bacillus timonensis*	NR_133024.1	99.4	JBDFMO000000000	*"Bacillus timonensis"*	GCF_902374785.1	83.0	85.3	26.7	87.3
*Paenibacillus canaveralius*	1P03SA^T^	EU977770	*Paenibacillus chitinolyticus*	NR_112053.1	99.1	JBDFMM000000000	*Paenibacillus chitinolyticus*	GCF_004117095.1	90.1	91.9	38.7	94.8
*Arthrobacter phoenicis*	1P04PC^T^	EU977744	*Pseudarthrobacter phenanthrenivorans*	NR_074770.2	98.2	JBDFMK000000000	*Arthrobacter oryzae*	GCF_003634095.1	83.0	80.2	24.6	N/A
*Microbacterium phoenicis*	1P06AB^T^	EU977652	*Microbacterium oleivorans*	NR_042262.1	99.8	CP154849	*Microbacterium paludicola*	GCF_001975955.2	86.0	88.3	29.4	91.8
*Bacillus kalamii*	1P06AnD^T^	PP475408	*Bacillus testis*	NR_144719.1	99.2	JBDFMI000000000	*"Bacillus testis"*	GCF_001243895.1	80.0	76.9	21.0	82.0
*Sphingomonas canaveralia*	1P06PA^T^	EU977746	*Sphingomonas prati*	NR_152092.1	96.8	JBDFMH000000000	*Sphingomonas jatrophae*	GCF_900113315.1	79.7	66.8	20.2	78.9
*Peribacillus phoenicis*	1P06PB^T^	EU977747	*Peribacillus frigoritolerans*	NR_115064.1	99.9	JBDFMF000000000	*Peribacillus frigoritolerans*	GCF_022394675.1	93.5	94.3	54.4	97.8
*Oceanobacillus phoenicis*	1P07AA^T^	EU977643	*Oceanobacillus iheyensis*	NR_075027.2	99.0	JBDFME000000000	*Oceanobacillus kimchii*	GCF_000340475.1	90.4	92.3	40.3	93.2
*Paenibacillus jepli*	1P07SE^T^	PP475409	*Paenibacillus hispanicus*	NR_152687.1	98.6	JBDFMD000000000	*Paenibacillus daejeonensis*	GCF_000378385.1	81.0	82.0	22.4	84.7
*Sphingomonas phoenicis*	1P08PE^T^	EU977752	*Sphingomonas roseiflava*	NR_117716.1	97.3	JBDFMC000000000	*Sphingomonas metalli*	GCF_014641735.1	83.0	77.4	23.7	N/A
*Microbacterium canaveralium*	1P10AE^T^	EU977655	*Microbacterium timonense*	NR_179660.1	98.4	JBDFMA000000000	*Microbacterium hydrothermale*	GCF_004854025.1	84.0	80.8	25.4	79.7
*Curtobacterium phoenicis*	1P10AnD^T^	EU977722	*Curtobacterium pusillum*	NR_042315.1	99.5	JBDFLZ000000000	*Curtobacterium luteum*	GCF_014646995.1	89.0	90.1	33.4	80.0
*Noviherbaspirillum phoenicis*	1P10PC^T^	EU977754	*Noviherbaspirillum aurantiacum*	NR_118040.1	99.7	JBDFLX000000000	*Noviherbaspirillum soli*	GCF_015352955.1	93.6	94.2	52.4	97.9
*Neobacillus phoenicis*	1P10SD^T^	EU977785	*Neobacillus bataviensis*	NR_036766.1	98.6	CP154847	*"Bacillus salipaludis"*	GCF_004358205.1	79.0	73.2	20.8	78.3
*Microbacterium jepli*	1P10UB^T^	EU977807	*Microbacterium timonense*	NR_179660.1	97.6	JBDFLW000000000	*Microbacterium lemovicicum*	GCF_003991875.1	93.0	94.2	47.5	95.4
*Robertmurraya phoenicis*	2P01SA^T^	PP475410	*Robertmurraya massiliosenegalensis*	NR_125590.1	99.1	CP154845	*Robertmurraya massiliosenegalensis*	GCF_000311725.1	90.8	92.2	41.8	92.0
*Microbacterium pratiae*	2P06AB^T^	EU977682	*Microbacterium arborescens*	NR_029265.1	99.9	CP154843	*Microbacterium oleivorans*	GCF_001887285.1	91.0	93.2	39.2	N/A
*Brevundimonas phoenicis*	2P2-tot-C^T^	PP475411	*Brevundimonas diminuta*	NR_040805.1	99.2	JBDFLN000000000	*Brevundimonas diminuta*	GCF_900445995.1	93.3	92.0	47.0	97.0
*Lysinibacillus canaveralius*	3P01SB^T^	EU977788	*Lysinibacillus odysseyi*	NR_025258.1	99.5	CP154840	*Lysinibacillus odysseyi*	GCF_001591965.1	84.0	87.6	28.1	88.2
*Neobacillus canaveralius*	3P2-tot-E-2^T^	PP475412	*Neobacillus niacin*i	NR_024695.1	99.3	JBDFLL000000000	*Neobacillus niacini*	GCF_001591505.1	87.0	87.8	33.4	90.6

### Phylogenomic analysis

The phylogenomic analysis based on the 16S rRNA gene, gyrB, and WGS was performed, and these novel organisms were placed in their respective phylogenetic trees to determine their precise taxonomic placement.

Members of *Actinomycetota* phylum showed varied ANI index similarities when compared to established species. The ANI index of *Agrococcus phoenicis* 1P02AA revealed a low similarity (79–86%) with already recognized *Agrococcus* species, with *Agrococcus carbonis* being the closest species at 86% ANI. However, based on the single-copy core genes, *Agrococcus baldri* was the closest species, with 85.63% ANI. Three strains from *Arthrobacter phoenicis* of the present study exhibited 100% similarity among themselves and were closely related to *Arthrobacter oryzae*, with 83% ANI. The species *Curtobacterium phoenicis* was closely related to *Curtobacterium* *luteum*, exhibiting an ANI similarity of 89%. The strains belonging to *Georgenia phoenicis* (1P01AC and 1P07AB) were 100% similar to each other and presented an ANI of 89% to the closest relative, *Georgenia satyanarayanai*. The species belonging to *Microbacterium* genus (*M.* *canaveralium*, *M. jepli*, *M.* *phoenicis*, and *M.* *pratiae)* presented ANI values ranging from 84 to 93% compared with their closest relatives.

Four novel species were identified as belonging to *Pseudomonadota* phylum. *Noviherbaspirillum phoenicis* were closely related to *Noviherbaspirillum soli*, exhibiting an ANI of 94%. The novel species *Brevundimonas phoenicis* comprising 18 strains, clustered together with 100% ANI and showed 93% ANI similarity with *Brevundimonas diminuta.* Similarly, the four strains of *Pseudomonas* *phoenicis* were grouped with 100% ANI and showed ~ 86% similarity with the closest species *Pseudomonas* *cremoricolorata.* The novel species *Sphingomonas canaveralia* was placed near *Sphingomonas jatrophae* with a 79% ANI, and *Sphingomonas phoenicis* was adjacent to *Sphingomonas metalli* with an 83% ANI.

Additionally, among the strains belonging to the *Bacillota* phylum, the *Alkalihalobacillus* and *Shouchella* genera were placed in the same phylogenomic tree due to their similarity. *Alkalihalobacillus phoenicis* 1P02AB was closest to *Alkalihalobacillus alcalophilus* with an ANI of 92%, while the strains of *Shouchella phoenicis* were similar among themselves and closest to *Shouchella hunanensis* with an ANI of 81%. The novel species *Bacillus* *jepli*, and *B.* *kalamii* were not closely related, with ANI value of 80%, and similar patterns were observed when compared with other strains of *Bacillus* genus (ANI ranging from 76 to 83%). *Lysinibacillus canaveralius* clustered with *Lysinibacillus odysseyi*, presenting an ANI of 84%, while *Lysinibacillus phoenicis* was closely related to *Lysinibacillus* *fusiformis* with an ANI of 85%. Two species of *Neobacillus*, *N.* *canaveralius* and *N.* *phoenicis*, were distant from each other, with an ANI of 79%. Phylogenetic analysis revealed that *N.* *canaveralius* is closer to *N.* *niacini*, with an ANI of 87%, while *N.* *phoenicis* is closer to *N.* *bataviensis*, with an ANI of 79%. The single species *Oceanobacillus phoenicis* presented a high ANI percentage with its closest relative, *Oceanobacillus* *kimchi* with ANI of 90%. The novel representatives of *Paenibacillus*, *P. jepli* and *P.* *canaveralius*, were closely related to *P. daejeonensis* (ANI 81%) and *P.* *chitinolyticus* (ANI 90%), respectively. Two strains of *Peribacillus phoenicis* clustered together with 100% ANI and showed 94% similarity to *Peribacillus frigoritolerans*, its closest relative. The species *Robertmurraya phoenicis* was similar to *Robertmurraya massiliosenegalensis*, with a 91% ANI.

**Fig. 1:**
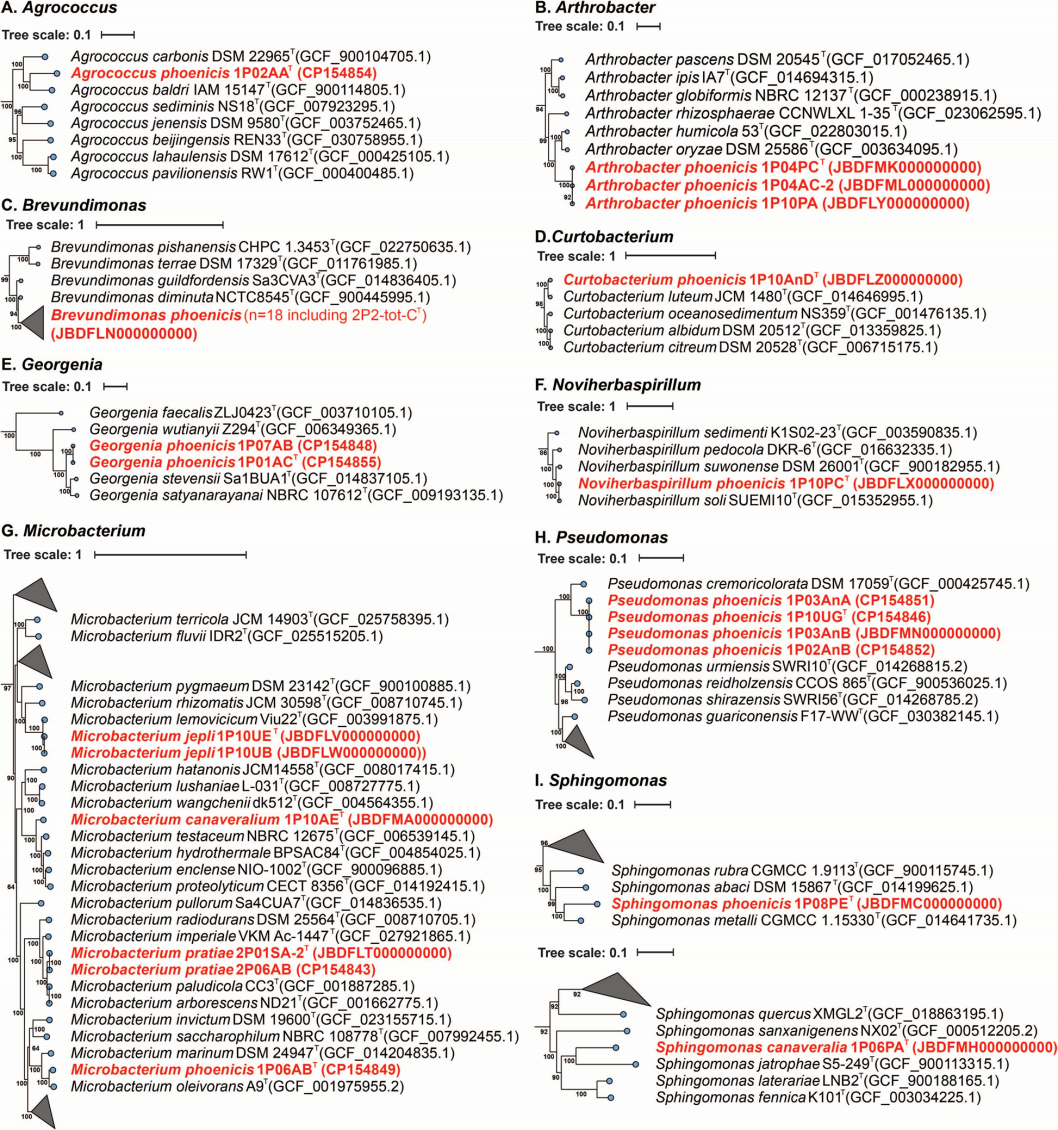
Phylogenetic tree of non-spore-forming bacteria (*n* = 38) spanning into nine genera from Phoenix spacecraft mission. Novel species are highlighted in red, and their corresponding NCBI accessions are provided. Bootstrap values (expressed as percentages) are indicated near the branches

**Fig. 2:**
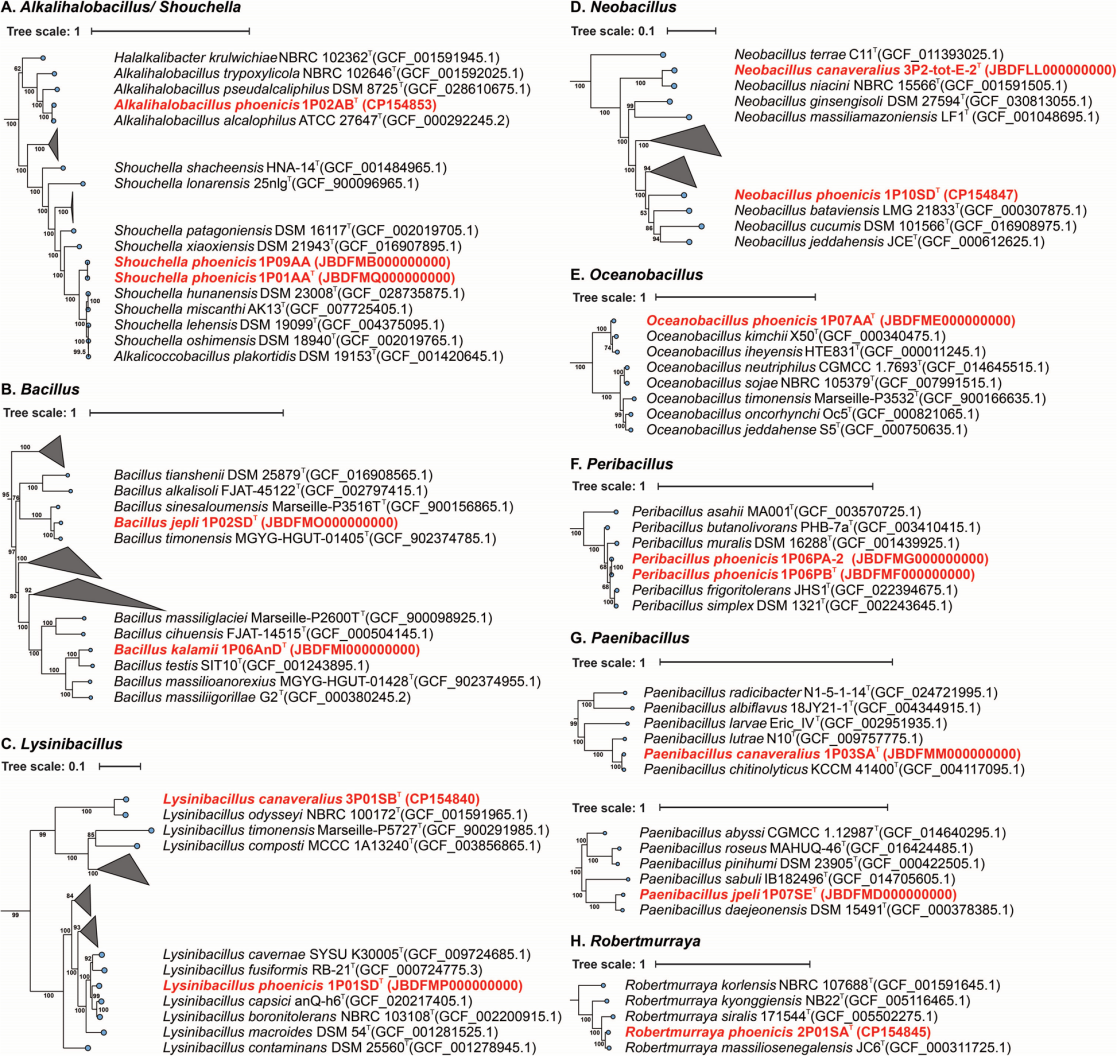
Phylogenetic tree of 15 novel strains of spore-forming bacteria from *Bacillaceae* family isolated from Phoenix spacecraft mission. Novel species are highlighted in red, and their corresponding NCBI accessions are provided. Bootstrap values (expressed as percentages) are indicated near the branches

To further validate the placement of the novel species within the bacterial tree of life, a phylogenetic tree was generated by comparing them with 4441 complete, non-anomalous representative genomes of bacteria (Supplementary Figure S3). The tree of life showed that these novel genomes are almost distributed across the entire spectrum, indicating that spacecraft assembly cleanrooms can harbor a wide range of bacterial diversity. Additionally, 17 phylogenetic trees were constructed at the genus level, with Fig. 1 representing non-spore-formers and Fig. 2 representing spore-formers.

### Morphological characterization

**Fig. 3:**
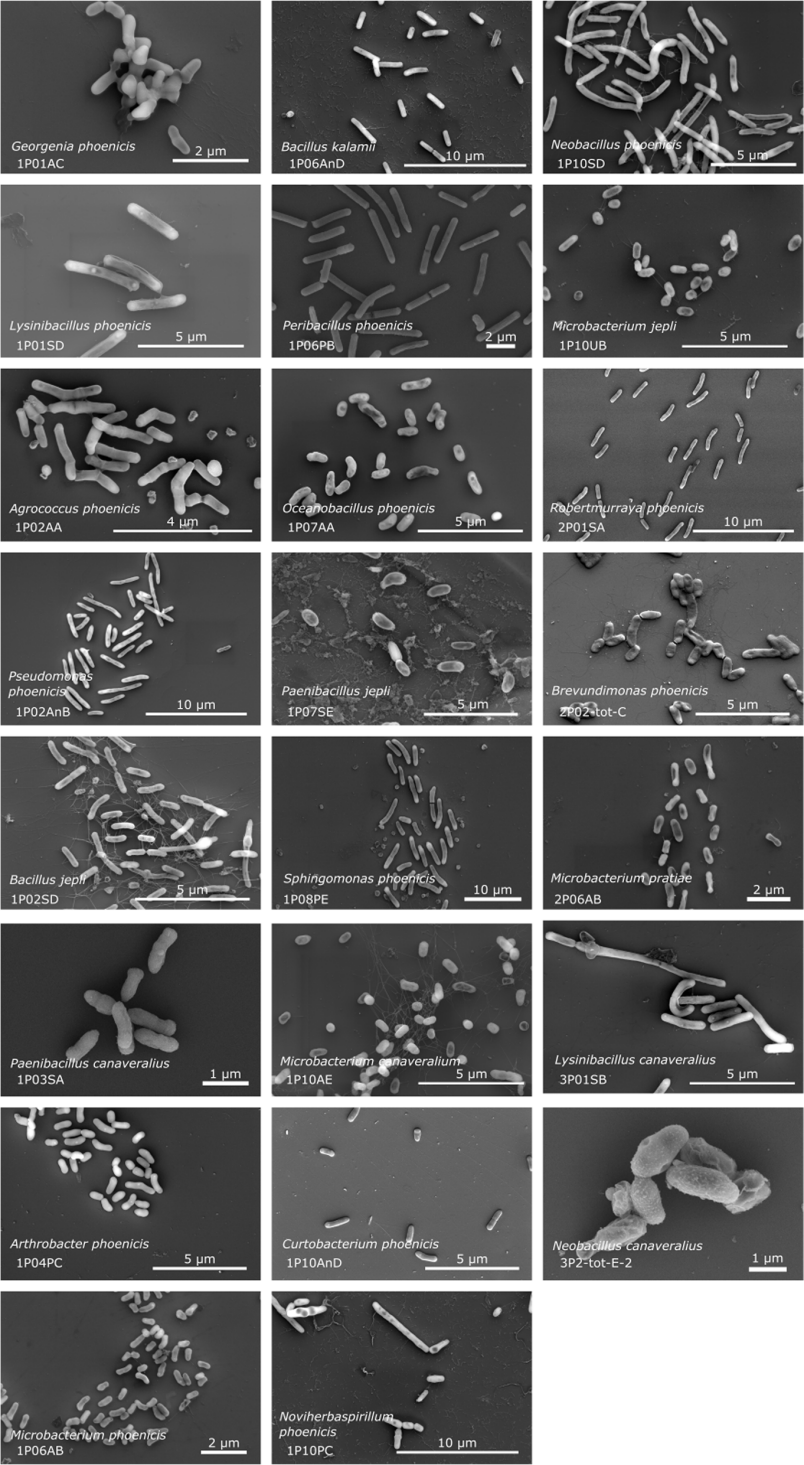
Scanning electron microscopy of the novel species isolated from the Phoenix spacecraft assembly cleanroom

To analyze the bacterial isolates in further detail, Gram staining was performed on each isolate. Of all the isolates, 77% were Gram-negative, while the rest (23%) were characterized as Gram-positive bacteria. For in-depth morphological characterization scanning electron microscopy (SEM) analysis was carried out for all the isolates characterized as novel species. Many of the bacterial cells exhibited round or rod-shaped morphologies, presenting either as single cells or in aggregation of multiple cells. The details of the microscopic characterization of each isolate are presented in Table 2, based on SEM images (Fig. 3) and Gram staining images (Supplementary Figure S4). The novel species etymologies are given in Table 2.

**Table 2:** Species epithet and etymology of the novel species described during this study

Novel species name proposed	Strain number (other culture collection numbers)	BioLog identification^a^	MALDI identification^b^	Cell characteristics	Spore formation	Gram stain characteristics	Species etymology
*Shouchella phoenicis*	1P01AA^T^ = DSM 118000^T^ =MTCC 13943^T^	No ID possible	No ID possible	Round-shaped; cells are aerobic, motile, and rod-shaped. Colony on TSA medium is beige, circular, entire margin, smooth, non-transparent, and raised.	Yes	Gram-positive	*Shouchella phoenicis* (phoe’ni.cis. L. gen. n. *phoenicis* of phoenix, isolated from the surface of the clean room where the Mars Phoenix spacecraft was assembled).
*Georgenia phoenicis*	1P01AC^T^ = DSM 117974^T^ = MTCC 13825^T^ = NRRL B-59271^T^	*Corynebacterium pilosum*	No ID possible	Round-shaped; cells are aerobic, motile, short rods (0.5 µm ± 0.2 µm in width and 0.5 µm in length). Colonies on R2A medium are light yellow, circular and opaque after incubation at 25 °C for 48 h.	No	Gram-positive	Georgenia phoenicis (phoe’ni.cis. L. gen. n. *phoenicis* of phoenix, isolated from the surface of the clean room where the Mars Phoenix spacecraft was assembled).
*Lysinibacillus phoenicis*	1P01SD^T^ = DSM 117981^T^ = MTCC 13636^T^	*Lysinibacillus boronitolerans*	*Lysinibacillus fusiformis*	Rod-shaped; cells are aerobic, motile, 2.5 µm ± 0.1 µm long and 0.5 µm in width. Colonies on R2A medium are white, circular and opaque after incubation at 25 °C for 48 h.	Yes	Gram-positive	*Lysinibacillus phoenicis* (phoe’ni.cis. L. gen. n. *phoenicis* of phoenix, isolated from the surface of the clean room where the Mars Phoenix spacecraft was assembled).
*Agrococcus phoenicis*	1P02AA^T^ = DSM 117973^T^ = NRRL B-59275^T^	No ID possible	No ID possible	Round-shaped; cells are aerobic, non-motile, short rods (0.3 µm in width and 0.9 ± 0.4 µm in length). Colonies on R2A medium are light yellow, circular and opaque after incubation at 25 °C for 48 h.	No	Gram-positive	*Agrococcus phoenicis* (phoe’ni.cis. L. gen. n. *phoenicis* of phoenix, isolated from the surface of the clean room where the Mars Phoenix spacecraft was assembled).
*Alkalihalobacillus phoenicis*	1P02AB^T^ = DSM 118404^T^ = MTCC 13926^T^ = NRRL 59276^T^	No ID possible	No ID possible	Rod-shaped cells, aerobic, and endospore forming and motile. Colony on TSA medium is beige to transparent color, circular, entire margin, smooth, and raised.	Yes	Gram-positive	*Alkalihalobacillus phoenicis* (phoe’ni.cis. L. gen. n. *phoenicis* of phoenix, isolated from the surface of the clean room where the Mars Phoenix spacecraft was assembled).
*Pseudomonas phoenicis*	1P02AnB^T^ = DSM 119702^T^ = MTCC 13927^T^	No ID possible	*Pseudomonas protoformans* (1.64)	Round-shaped; cells are aerobic, motile, with 1.8 ± 0.5 µm in length and 0.5 ± 0.1 µm in width. Colonies on R2A medium are light yellow, circular and opaque after incubation at 25 °C for 48 h.	No	Gram- negative	*Pseudomonas phoenicis* (phoe’ni.cis. L. gen. n. *phoenicis* of phoenix, isolated from the surface of the clean room where the Mars Phoenix spacecraft was assembled).
*Bacillus jepli *	1P02SD^T^ = DSM 117993^T^ = MTCC 13928^T^ = NRRL 59278^T^	No ID possible	No ID possible	Round-shaped; cells are aerobic, motile, with 1.3 ± 0.5 µm in length and 0.4 ± 0.1 µm in width. Colonies on R2A medium are light beige, circular and opaque after incubation at 25 °C for 48 h.	Yes	Gram-positive	*Bacillus jepli (jep’li. N.L. gen. n. jepli, arbitrary name derived from the abbreviation JPL, meaning of or pertaining to the NASA’s Jet Propulsion Laboratory, where the type strain of the species was isolated).*
*Paenibacillus canaveralius*	1P03SA^T^ = DSM 117982^T^ = MTCC 13637^T^	*Paenibacillus assamensis*	No ID possible	Rod-shaped; cells are aerobic, motile, short rods (0.4 µm ± 0.1 µm in width and 0.9 ± 0.2 µm in length). Colonies on R2A medium are white, irregular and opaque after incubation at 25 °C for 48 h.	Yes	Gram-positive	*Paenibacillus canaveralius (ca.na.ve.ra’li.us. N.L. masc. adj. canaveralius pertaining to (Cape) Canaveral, isolated from walls and floors of the Kennedy Space Center at Cape Canaveral).*
*Arthrobacter phoenicis*	1P04PC = DSM 117984^T^ = MTCC13669^T^	*Arthobacter ilicis*	No ID possible	Rod-shaped; cells are aerobic, non-motile, short rods (0.5 µm in width and 1.0 ± 0.2 µm in length). Colonies on R2A medium are light beige, circular and opaque after incubation at 25 °C for 48 h.	No	Gram-negative	*Arthrobacter phoenicis* (phoe’ni.cis. L. gen. n. *phoenicis* of phoenix, isolated from the surface of the clean room where the Mars Phoenix spacecraft was assembled).
*Microbacterium phoenicis*	1P06AB = DSM 117975^T^	No ID possible	No ID possible	Round-shaped; cells are aerobic, non-motile, short rods (0.2 µm ± 0.1 µm in width and 0.5 ± 0.2 µm in length). Colonies on R2A medium are dark beige, circular and opaque after incubation at 25 °C for 48 h.	No	Gram-positive	*Microbacterium phoenicis* (phoe’ni.cis. L. gen. n. *phoenicis* of phoenix, isolated from the surface of the clean room where the Mars Phoenix spacecraft was assembled).
*Bacillus kalamii*	1P06AnD = DSM 117991^T^ = MTCC 13638^T^	No ID possible	No ID possible	Rod-shaped; cells are aerobic, motile, with 2.1 ± 0.7 µm in length and 0.6 ± 0.1 µm in width. Colonies on R2A medium are light beige, circular and opaque after incubation at 25 °C for 48 h.	Yes	Gram-positive	*Bacillus kalamii* (ka.lam′i.i. N.L. gen. n. kalamii referring to Abdul Kalam, a well-known scientist who advanced space research in India).
*Sphingomonas canaveralia*	1P06PA^T,C^	No ID possible	No ID possible	Cells are aerobic, non-motile, short rods or ovoid. Colonies on R2A medium are bright yellow, small, circular, and entire margin, smooth, and raised after incubation at 25 °C for 2 to 7 days.	No	Gram-negative	*Sphingomonas canaveralia (ca.na.ve.ra’li.a. N.L. fem. adj. canaveralia pertaining to (Cape) Canaveral, isolated from walls and floors of the Kennedy Space Center at Cape Canaveral).*
*Peribacillus phoenicis*	1P06PB^T^ = DSM 117992^T^ = MTCC 13942^T^ = NRRL 59293^T^	No ID possible	*Oceanobacillus kimchii* (1.78)	Rod-shaped; cells are aerobic, motile, with 3.0 ± 1.1 µm in length and 0.6 ± 0.1 µm in width. Colonies on R2A medium are light beige, circular and opaque after incubation at 25 °C for 48 h.	Yes	Gram-positive	*Peribacillus phoenicis* (phoe’ni.cis. L. gen. n. *phoenicis* of phoenix, isolated from the surface of the clean room where the Mars Phoenix spacecraft was assembled).
*Oceanobacillus phoenicis*	1P07AA = DSM 118405^T^	No ID possible	No ID possible	Rod-shaped; cells are aerobic, motile, short rods (0.4 µm ± 0.1 µm in width and 0.9 ± 0.3 µm in length). Colonies on R2A medium are light beige, circular and opaque after incubation at 25 °C for 48 h.	Yes	Gram-positive	*Oceanobacillus phoenicis* (phoe’ni.cis. L. gen. n. *phoenicis* of phoenix, isolated from the surface of the clean room where the Mars Phoenix spacecraft was assembled).
*Paenibacillus jepli *	1P07SE^T^ = DSM 118005^T^ = MTCC 13640^T^	*Paenibacillus assamensis*	No ID possible	Rod-shaped; cells are aerobic, motile, 1.4 ± 0.3 µm in length and 0.7 µm ± 0.1 µm in width. Colonies on R2A medium are light beige, circular and opaque after incubation at 25 °C for 48 h.	Yes	Gram-positive	*Paenibacillus jepli (jep’li. N.L. gen. n. jepli, arbitrary name derived from the abbreviation JPL, meaning of or pertaining to the NASA’s Jet Propulsion Laboratory, where the type strain of the species was isolated.*
*Sphingomonas phoenicis*	1P08PE^T^ = DSM 119703^T^	No ID possible	No ID possible	Rod-shaped; cells are aerobic, non-motile, 1.6 ± 0.5 µm in length and 0.5 µm ± 0.1 µm in width. Colonies on R2A medium are orangeish, circular and opaque after incubation at 25 °C for 48 h.	No	Gram-negative	*Sphingomonas phoenicis* (phoe’ni.cis. L. gen. n. *phoenicis* of phoenix, isolated from the surface of the clean room where the Mars Phoenix spacecraft was assembled).
*Microbacterium canaveralium*	1P10AE = DSM 117987^T^ = MTCC 13670^T^	No ID possible	No ID possible	Round-shaped; cells are aerobic, non-motile, short rods (0.6 µm ± 0.1 µm in width and 1.1 ± 0.4 µm in length). Colonies on R2A medium are yellow, circular and opaque after incubation at 25 °C for 48 h.	No	Gram-positive	*Microbacterium canaveralium (ca.na.ve.ra’li.um. N.L. neut. adj. canaveralium pertaining to (Cape) Canaveral, isolated from walls and floors of the Kennedy Space Center at Cape Canaveral).*
*Curtobacterium phoenicis*	1P10AnD = DSM 117985^T^ = MTCC 13639^T^	*Curtobacterium citreum*	No ID possible	Round-shaped; cells are aerobic, motile, short rods (0.4 µm in width and 1.0 ± 0.2 µm in length). Colonies on R2A medium are yellow, circular and opaque after incubation at 25 °C for 48 h.	No	Gram-positive	*Curtobacterium phoenicis* (phoe’ni.cis. L. gen. n. *phoenicis* of phoenix, isolated from the surface of the clean room where the Mars Phoenix spacecraft was assembled).
*Noviherbaspirillum phoenicis*	1P10PC^T^ = DSM 118057^T^ = MTCC 13944^T^	No ID possible	No ID possible	Round-shaped; cells are aerobic, motile, 1.4 ± 0.6 µm in length and 0.6 µm ± 0.1 µm in width. Colonies on R2A medium are light beige, circular and opaque after incubation at 25 °C for 48 h.	No	Gram-negative	*Noviherbaspirillum phoenicis* (phoe’ni.cis. L. gen. n. *phoenicis* of phoenix, isolated from the surface of the clean room where the Mars Phoenix spacecraft was assembled).
*Neobacillus phoenicis*	1P10SD = DSM 118030^T^ = MTCC 13641^T^	*Bacillus sp.*	No ID possible	Rod-shaped; Cells are aerobic, motile, 1.8 ± 1.0 µm in length and 1.1 µm ± 1.1 µm in width. Colonies on R2A medium are light beige, circular and opaque after incubation at 25 °C for 48 h.	Yes	Gram-positive	*Neobacillus phoenicis* (phoe’ni.cis. L. gen. n. *phoenicis* of phoenix, isolated from the surface of the clean room where the Mars Phoenix spacecraft was assembled).
*Microbacterium jepli *	1P10UB^T,C^	No ID possible	No ID possible	Round-shaped; cells are aerobic, non-motile, short rods (0.4 µm ± 0.1 µm in width and 0.7 ± 0.2 µm in length). Colonies on R2A medium are white, circular and opaque after incubation at 25 °C for 48 h.	No	Gram-positive	*Microbacterium jepli (jep’li. N.L. gen. n. jepli, arbitrary name derived from the abbreviation JPL, meaning of or pertaining to the NASA’s Jet Propulsion Laboratory, where the type strain of the species was isolated.*
*Robertmurraya phoenicis*	2P01SA = DSM 118031^T^ = MTCC 13642^T^	*Brevibacillus borstelensis*	*Robertmurraya massiliosenegalensis (1.75)*	Rod-shaped; cells are aerobic, motile, 2.1 µm ± 0.5 µm long and 0.5 µm ± 0.1 µm in width. Colonies on R2A medium are light white, circular and opaque after incubation at 25 °C for 48 h.	Yes	Gram-positive	*Robertmurraya phoenicis* (phoe’ni.cis. L. gen. n. *phoenicis* of phoenix, isolated from the surface of the clean room where the Mars Phoenix spacecraft was assembled).
*Microbacterium pratiae*	2P06AB = DSM 117976^T^ = MTCC 13671^T^	*Microbacterium arboescens*	No ID possible	Round-shaped; cells are aerobic, non-motile, short rods (0.4 µm ± 0.1 µm in width and 1.0 ± 0.4 µm in length). Colonies on R2A medium are dark beige, circular and opaque after incubation at 25 °C for 48 h.	No	Gram-positive	*Microbacterium pratiae* (pra’ti’ae. N.L. gen. fem. n. *pratiae* referring to Dr. Lisa Pratt, a biogeochemist and astrobiologist who previously served as the Planetary Protection Officer for NASA).
*Brevundimonas phoenicis*	2P2-tot-C^T^ = DSM 118058^T^ = MTCC 13894^T^	*Brevundimonas diminuta*	No ID possible	Round-shaped; cells are aerobic, motile, short rods (0.6 µm ± 0.1 µm in width and 1.2 ± 0.4 µm in length). Colonies on R2A medium are light beige, circular and opaque after incubation at 25 °C for 48 h.	No	Gram-negative	*Brevundimonas phoenicis* (phoe’ni.cis. L. gen. n. *phoenicis* of phoenix, isolated from the surface of the clean room where the Mars Phoenix spacecraft was assembled).
*Lysinibacillus canaveralius*	3P01SB = DSM 118058^T^ = MTCC 13643^T^	*Lysinibacillus* sp.	No ID possible	Rod-shaped; cells are aerobic, motile, 1.7 µm ± 0.7 µm long and 0.4 µm ± 0.1 µm in width. Colonies on R2A medium are white, circular and opaque after incubation at 25 °C for 48 h.	Yes	Gram-positive	*Lysinibacillus canaveralius (ca.na.ve.ra’li.us. N.L. masc. adj. canaveralius pertaining to (Cape) Canaveral, isolated from walls and floors of the Kennedy Space Center at Cape Canaveral).*
*Neobacillus canaveralius*	3P2-tot-E-2^T^ = DSM 118040^T^ = MTCC 13929^T^	No ID possible	No ID possible	Rod-shaped; cells are aerobic, motile, 2.3 ± 1.0 µm in length and 1.0 µm ± 0.5 µm in width. Colonies on R2A medium are light beige, circular and opaque after incubation at 25 °C for 48 h.	Yes	Gram-positive	*Neobacillus canaveralius (ca.na.ve.ra’li.us. N.L. masc. adj. canaveralius pertaining to (Cape) Canaveral, isolated from walls and floors of the Kennedy Space Center at Cape Canaveral).*

^a^Methods followed as per established procedure [15]

^b^Methods followed as per established procedure [16]. The number in paranthesis denotes MALDI ID index #. Anything >2.0 is considered as identified species

^c^Culture collection is processing the viability of the strains submitted

### Phenotypic characterization

The phenotypic identification results obtained using the BioLog GenIII system (Supplementary Table S2), and Matrix-Assisted Laser Desorption/Ionization (MALDI) assay are presented in Table 2. Both assays, which depend on databases of known microorganisms (mostly clinical origin), were unable to accurately identify the novel species. In the BioLog system, only 11 out of 26 novel species were identified to the genus level (Supplementary Table S2), while the MALDI profile could assign only 4 out of 26 species to their genera and none were identified at the species. The majority of species were classified as “no identification,” highlighting the limitations of phenotype-based methods for identifying novel species. This further emphasizes the importance of WGS-based phylogeny, which provides greater accuracy, reproducibility, and reliability in microbial classification. Additionally, the novel species identified in this study have been deposited in two culture collections, with their respective accession numbers listed in Table 2.

### Persistence of novel species

**Fig. 4:**
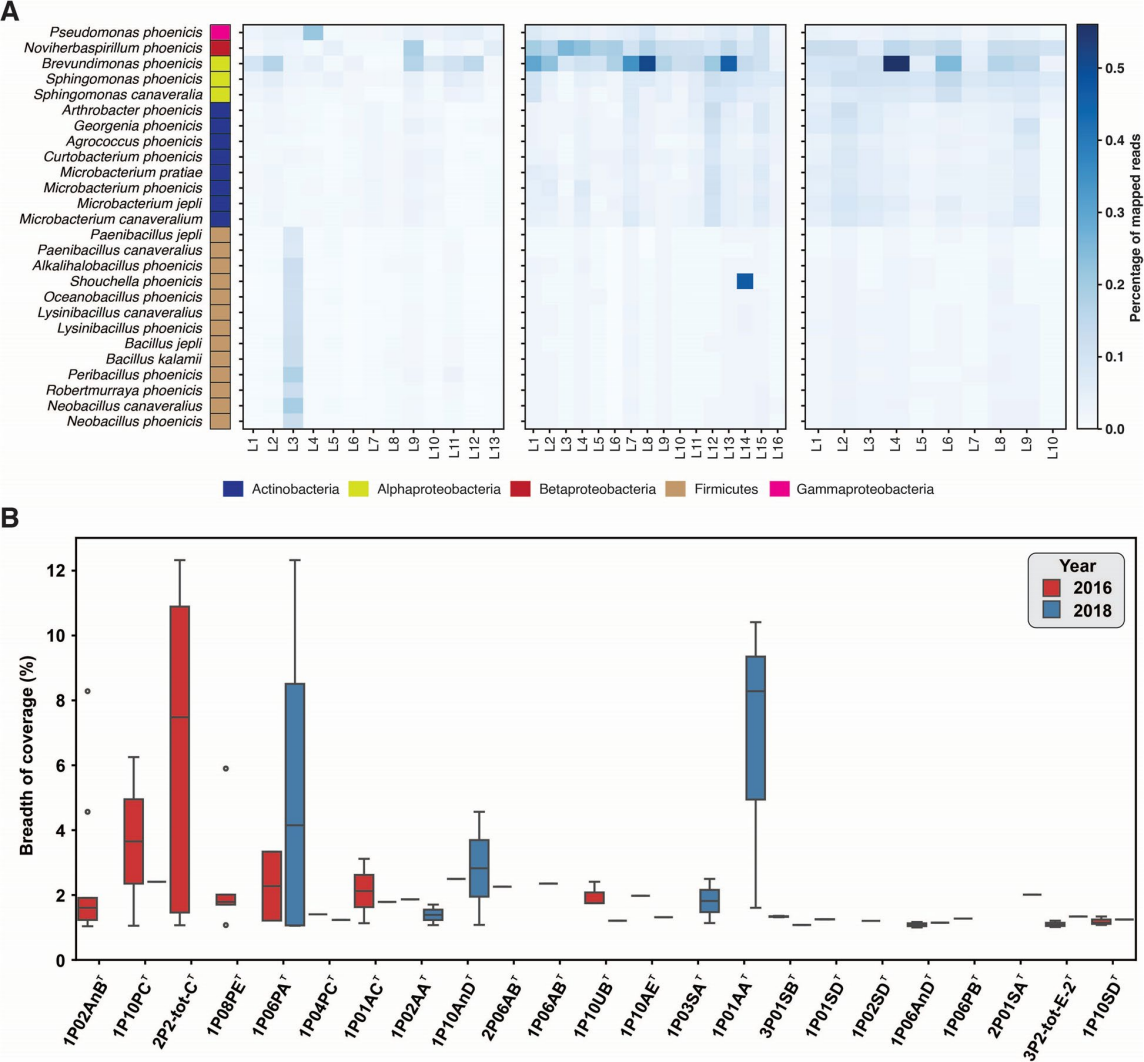
Metagenomic read mapping to novel isolates from NASA cleanrooms, highlighting temporal and spatial dynamics. **A** Spatial distribution of mapped reads across 26 novel species, showing distinct signatures between spore-forming and non-spore-forming bacteria in different NASA cleanroom locations. **B** Box plots illustrating the breadth of coverage (> 1%) of consensus genomes constructed from mapped reads aligned to 23 novel species (out of 26). Reads were collected from cleanrooms at SAF JPL and KSC-PHSF in 2016 (red) and KSC-PHSF in 2018 (blue)

Quality-filtered shotgun metagenomic reads were mapped onto 26 isolated novel species to assess their abundance based on the fraction of mapped reads and coverage breadth. Non-spore-formers had significantly more reads than spore-formers (Fig. 4A). Due to the limited proportion of mapped reads to novel species (< 1%), a read assembly was conducted to assess coverage breadth against isolated genomes. The average coverage breadth ranged from 0.0007 to 64.4% in JPL-SAF during 2016, from 0.00045 to 3.93% in JPL-SAF during 2018, and from 0.0004 to 6.8% in KSC-PHSF during 2018 (Supplementary Table S3). Using a 1% cutoff, the distribution of coverage breadth for novel species showing > 1% coverage (*n* = 23 species) in at least one sample is plotted in Fig. 4B. *B.* *phoenicis* demonstrated the highest mapping percentage, an anomaly, comprising 64.4% of total reads in a sample from location 9 in JPL-SAF. Additionally, *M.* *jepli* and *G.* *phoenicis* were present in more samples (*n* = 108) with > 1% coverage, followed by *P.* *phoenicis* (*n* = 105) and *A.* *phoenicis* (*n* = 104). Furthermore, three novel species *(A.* *phoenicis*, *O. phoenicis*, and *P. jepli*) were < 1% in their abundance in any of the samples and are not shown in Fig. 4B. This indicates that none of these 26 novel species dominate the cleanrooms and might be rare.

The methodologies employed in this study to retrieve MAGs previously yielded 42 MAGs from International Space Station (ISS) environmental samples, which are copiotrophic and primarily composed of human-associated known species [17–19] as well as novel, yet-to-be-described environmental species [20]. However, no MAGs were recovered from any of the cleanroom samples (> 500 samples) including 164 samples analyzed in this study via shotgun metagenomics, emphasizing the extreme oligotrophic nature of cleanroom environments and the challenges associated with metagenomic assembly in such low-biomass conditions.

### Functional characterization

Putative functions of the 26 novel bacterial species were annotated using Prokka and COG-classifier. A total of 212,520 CDS with 3807 distinct COG annotations were identified (Supplementary Table S4). Among the annotated subsystems, the top categories based on average gene counts included amino acids transport and metabolisms (259 genes), followed by transcription (232 genes), translation, ribosomal structure and biogenesis (229 genes), and carbohydrate transport and metabolism (225 genes). Further analysis of these organisms from the Phoenix spacecraft mission revealed that, on average, they possessed 74 genes predicted for defense mechanisms, primarily related to resistance to antibiotics and toxic compounds, and invasion and intracellular resistance.

**Fig. 5:**
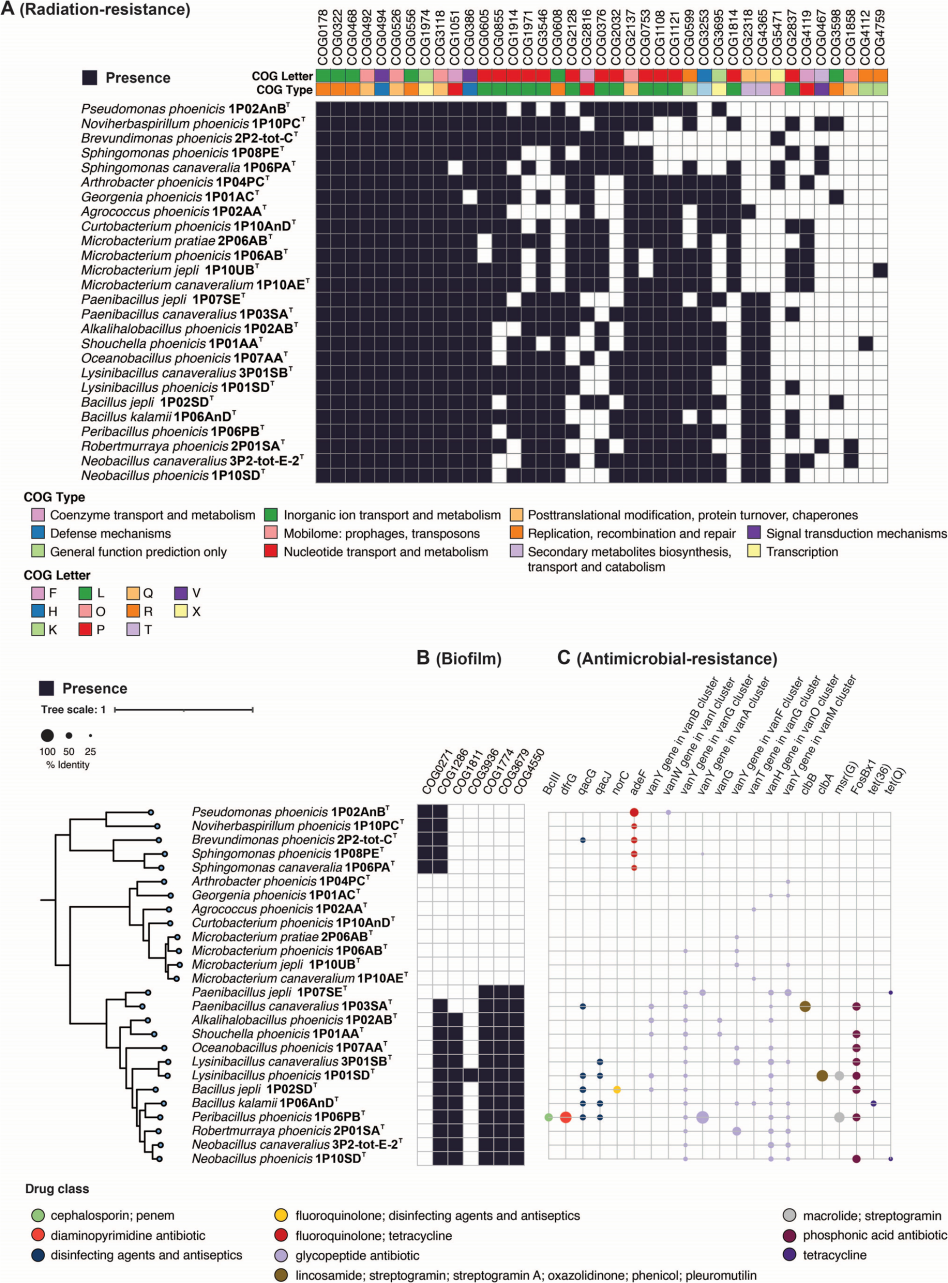
Functional insights into novel species from NASA cleanrooms. **A** Presence of radiation resistance COGs (from Pal et al. [21]) in the 26 novel species, revealing their genetic potential for radiation resilience. **B** Presence of biofilm-associated COGs in the novel bacterial species. **C**. Presence of antimicrobial-resistance genes and similarity with the drug class

Key genes potentially related to radiation resistance were observed across different bacterial isolates (Fig. 5A). The COG3253 proteins that were responsible for enhanced membrane transport and signaling under radiation were present in all spore-formers (*n* = 13) and eight novel actinobacterial species during this study. COG0608 genes, highlighting their role in DNA repair, were absent in all eight actinobacterial species but present in 18 other novel species. COG1108 genes, related to transcription regulation under radiation stress, were present in all novel species except alpha- and beta-proteobacteria (*n* = 24). COG1971 proteins involved in DNA repair after radiation exposure were found in all 13 spore-formers and five out of 13 non-spore-forming novel species. COG2318 proteins, associated with DNA repair and stress responses, were identified in spore-formers and *A.* *phoenicis*. COG4365 genes, responsible for increased radiation resistance, were present in all spore-formers but absent in other novel species. The involvement of COG4119 proteins in nucleotide excision repair pathways was reported in *Bacillus subtilis*, and in this investigation, this protein was present only in *N.* *canaveralius* whereas 12 other novel spore-formers lacked it.

The KMAP approach was used to recover the dataset of proteins of interest (POIs) from the novel species (Supplementary Figure S4A). While exploring the metabolic potential of these novel species, various noteworthy observations were made, including the annotation of several hundred proteins in different application categories. Notably, higher numbers of proteins related to bioprocess engineering, medicine and pharmaceuticals, and analytics were observed, particularly those involved in synthesis, drug development, agriculture, the food industry, and molecular biology. POIs relevant to withstanding extremophilic conditions (such as high temperature and alkalinity) were also identified.

### Biofilm formation

The biofilm-associated COG proteins observed across various bacterial isolates are depicted in Fig. 5B. The DNA-binding global transcriptional regulator BolA, which affects cell shape, cell division, and biofilm formation (COG0271), was identified exclusively in proteobacterial members (5 species; 25 strains). Like BolA, the colicin V production accessory protein CvpA, a regulator of purF expression and biofilm formation (COG1286), was also present in proteobacterial members but absent in non-spore-forming species. Conversely, all novel spore-formers (12 species; 14 strains) except *Pa.* *jepli* contained COG1286. The membrane protein YqgA (COG1811), associated with biofilm formation, was found in most spore-forming members but was absent in both *Paenibacillus* species and all non-spore-forming members during this study. Membrane-bound acyltransferase YfiQ (COG3936), involved in biofilm formation and previously found in Yersinia pestis, was present only in *L.* *phoenicis* and not in any other 25 novel species identified in this research. A group of functionally related cell fate molecular regulators that controlled sporulation, competence, and biofilm development processes and events through modulation of gene and protein expression, such as COG1774 (YaaT); COG3679 (YlbF, YheA/YmcA); and COG4550 (YmcA, YheA/YmcA), was detected in all spore-formers but was absent in non-spore-forming species during this study.

### Antimicrobial resistance

Several AMR gene families were identified across the genomes, indicating resistance to ten distinct drug classes, with a predominance for fluoroquinolones, tetracyclines, disinfecting agents/antiseptics, phosphonic acids, and glycopeptides (Fig. 5C). The 53 genomes exhibited potential resistance to vancomycin and tetracycline antibiotics (Supplementary Table S5). The species *B.* *canaveralius*, *B. jepli*, *B. phoenicis*, *L. phoenicis*, *L. canaveralius*, *P. canaveralius*, *P. jepli*, *R. phoenicis*, *N. canaveralius*, and both strains of *Pe. phoenicis* (IP06PA-2 and 1P06PB) presented a higher amount of resistance genes. In terms of antibiotic resistance, five mechanisms were identified: the most common was antibiotic efflux, followed by antibiotic target alteration, antibiotic inactivation, and less commonly, antibiotic target protection and antibiotic target replacement. Overall, the genomic mining predicted the presence of 21 AMR genes, however, phenotypic investigation is necessary to validate the mechanisms.

### Biosynthetic gene clusters

In this study, we detected 138 distinct gene clusters that did not share any similarities to the Minimum Information about a Biosynthetic Gene Cluster (MIBiG) database [22]. We identified 14 gene clusters with 100% resemblance to existing clusters, including alkylresorcinol, bacillibactin, bacillopaline, carotenoid, ε-poly-L-lysine, and zeaxanthin (Supplementary Table S6). However, several partial biosynthetic gene clusters (BGC) were also found. We detected 19, 34, and 68 partial clusters with > 50%, > 25%, and < 25% similarity to known MIBiG clusters.

**Fig. 6:**
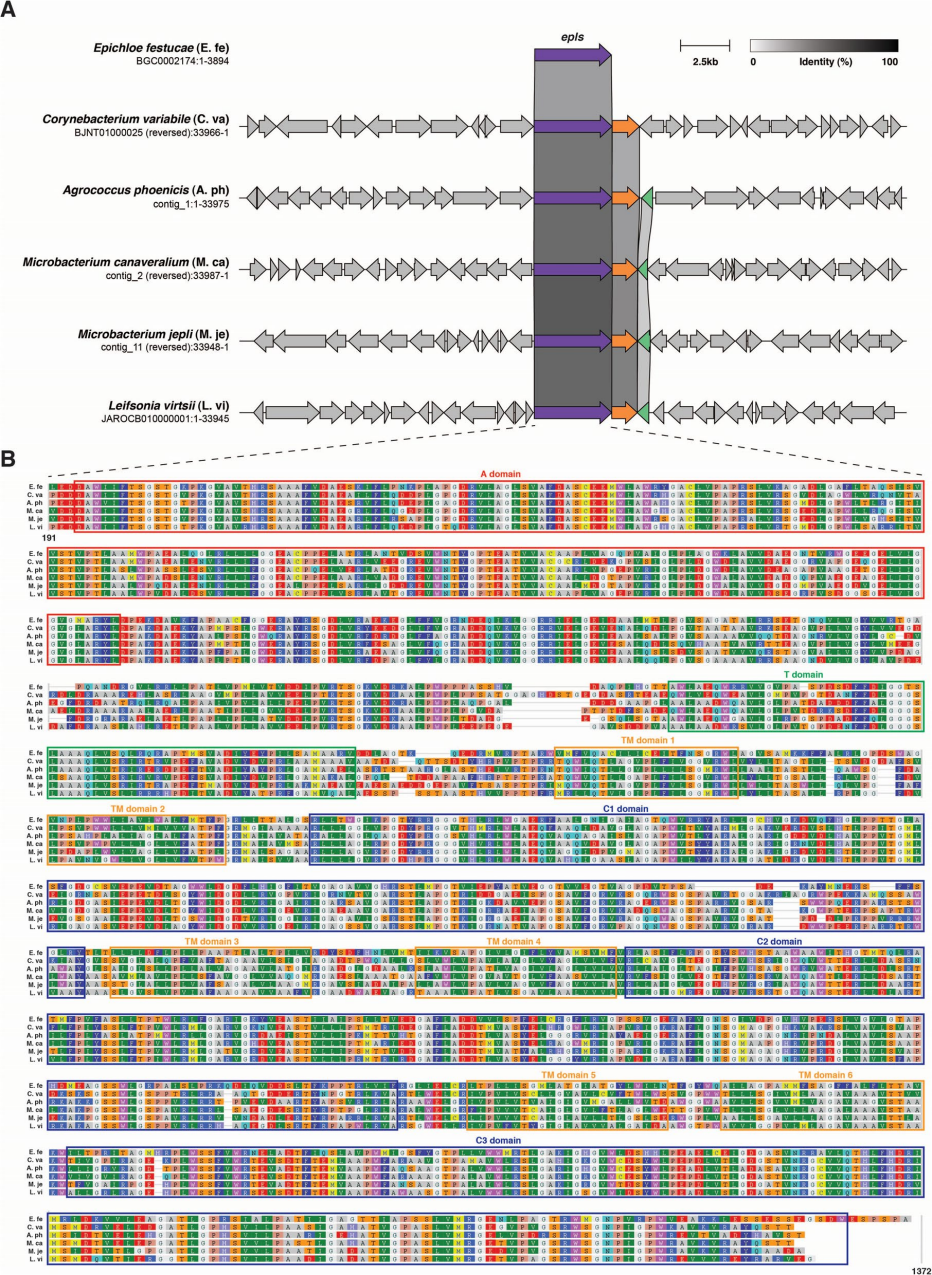
Comparative analysis of ε-poly-L-lysine synthetase (epls) in novel species. **A** ε-poly-L-lysine gene cluster comparison in *Epichloe festucae* (fungal producer), *Corynebacterium* *variabile* (bacterial producer), and three novel species from this study (*Agrococcus phoenicis*, *Microbacterium canaveralium*, *Microbacterium jepli*) and *Leifsonia virtsii* (isolated from ISS) show conserved gene cluster architecture. **B** Protein sequence alignment of ε-poly-L-lysine synthetase enzymes from these organisms exhibits conserved domains, including NRPS adenylation (A), thiolation (T), transmembrane (TM), and C-terminal tandem domains (C1, C2, C3)

A BCG analysis revealed 11 cluster types across 26 novel species, with T3PKS and terpene clusters being the most abundant (Supplementary Figure S4B). *P.* *jepli* 1P07SE and *S.* *phoenicis* 1P01AA exhibited the highest number of BGCs, with 12 and 10 BGCs each, respectively. BGCs from isolates showing > 80% similarity with known gene clusters, including alkylresorcinol, carotenoid, ε-poly-L-lysine, and paeninodin, were observed in 17 isolates (Supplementary Table S6). The ε-poly-L-lysine, known for its wide-spectrum inhibitory activity, heat stability, and biodegradability as a food preservative, was identified in three species (*A.* *phoenicis*, *M.* *canaveralium*, *M.* *jepli*) with 100% similarity. A gene cluster neighborhood comparison of ε-poly-L-lysine with known producers revealed functional ε-poly-L-lysine synthetase genes. Protein sequence comparison showed 48% identity with the fungal producer *Epichloe festucae* and around 67% identity with the bacterial producer *Corynebacterium* *variabile*, with the highest 70.8% identity in *M.* *canaveralium* (Fig. 6). Domain analysis indicated conserved non-ribosomal peptide synthetases adenylation (A) and thiolation (T) domains, six transmembrane (TM) domains, and three C-terminal tandem domains, crucial for substrate binding and lysine polymerization. This suggests potential for producing ε-poly-L-lysine, effective against foodborne pathogens like *E. coli* O157:H7, *Listeria monocytogenes*, *Staphylococcus aureus*, and *Serratia marcescens*.

## Discussion

Several factors contributed to the higher percentage of novel cultivable species (~ 25%; 53 out of 215 strains) retrieved from cleanrooms compared to 6 to 12% in natural environments [23, 24]. Studies demonstrated that extreme and controlled environments (low number of particles and stringent decontamination processes) might select unique microbial communities capable of thriving under harsh conditions (low-nutrient, desiccation, exposure to residual disinfectants, etc.). This eliminates many transient, dominant microbes while favoring those with high resistance to stressors and potentially driving microbial speciation and adaptation [25–29]. This process does not inherently generate novel species but creates conditions where rare, stress-tolerant microorganisms can persist, survive long-term, and become more detectable through cultivation. In cleanrooms, for instance, traditionally spore-formers are often reported. However, non-spore-formers such as *Arthrobacter*, *Brevundimonas*, *Georgenia*, *Microbacterium*, and *Pseudomonas* species, which can survive in oligotrophic, arid, and radiation conditions [30–34], should also be considered when setting bioburden requirements for future NASA missions. Additionally, the isolation of spore-formers like *Peribacillus* and *Shouchella* species, which require different cultural conditions compared to *Bacillus* species, underscores the importance of WGS in characterizing yet-to-be-recognized cultivable microbial species [35, 36]. Research on microbial isolates from the Atacama Desert further supported the notion that oligotrophic conditions and unique environmental pressures led to the discovery of more novel microbial taxa [37, 38]. The comprehensive genome analysis of the novel species revealed the presence of already established/peer-reviewed genetic adaptations that enable bacteria to survive extreme conditions, including genes responsible for resistance to radiation, desiccation, and other environmental stressors.

Experimental studies on *HemQ* (COG3253), also known as coproheme decarboxylase/chlorite dismutase, have demonstrated its significant role in coenzyme transport and metabolism, as well as inorganic ion transport and metabolism. In gram-positive bacteria (*Bacillota* and *Actinomycetota*) *HemQ* plays an essential role and has been associated with respiration, detoxification of reactive oxygen (ROS) and nitrogen species, gas sensing, and transport [39], a crucial property for stress survivability. This linkage (COG3253) was observed in all spore-formers (*n* = 13) and eight novel actinobacterial species in this study. Knockout experiments of COG0608 genes resulted in increased radiation sensitivity, demonstrating their role in DNA repair [40]. These genes were absent in all eight actinobacterial species found in this study but present in 18 other novel species. Except in alpha- and beta-proteobacteria, all other novel species (*n* = 24) exhibited COG1108 genes, which are related to transcription regulation under radiation stress, potentially confirming their protective role [41]. All 13 spore-formers and five out of 13 non-spore-forming novel species exhibited the presence of COG1971 proteins involved in DNA repair after radiation exposure, as reported to be upregulated in *D.* *radiodurans* [42]. Spore-formers and *A.* *phoenicis* had COG2318 proteins, which were experimentally proved to respond to radiation using a transcriptomic study in *D.* *radiodurans*, indicating potential roles in DNA repair and stress responses. All spore-formers, but not other novel species, exhibited the presence of COG4365 genes that were shown to be responsible for increased radiation resistance, confirming their potential role in DNA repair [43]. Radiation exposure studies in *B.* *subtilis* confirmed the involvement of COG4119 proteins in nucleotide excision repair pathways [44]. However, the absence of COG4119 proteins in 12 out of 13 spore-forming novel species in this study requires further investigation. Despite rigorous decontamination procedures, microbes possessing these traits likely contribute to their persistence in cleanroom environments.

The metagenome analysis, which aimed to correlate the persistence of novel microbes within the assembly facility after more than a decade of their isolation, revealed that these novel bacterial species were rare microbial species due to their low incidence in shotgun metagenomes and the overall breadth of coverage for their genomes. The genomes identified in this study have not been previously reported, and their low abundance in cleanroom metagenomes suggests they are rare members of these environments. It is hypothesized that their presence in cleanrooms is due to selective pressures that favor extremotolerant microbes, but their occurrence in other oligotrophic environments cannot be ruled out. Given the computational constraints of screening all publicly available metagenomes, our analysis was limited to datasets from the environments where these strains were originally isolated. Expanding such analyses to other low-nutrient, high-stress habitats could provide further insights into their ecological distribution and potential ubiquity in extreme environments. Although individually rare, members of these novel bacterial communities collectively might have played crucial roles in ecosystem functioning and stability, including nutrient cycling, decomposition, and symbiotic interactions, potentially leading to the discovery of novel bioactive compounds, enzymes, and metabolic pathways [45–47].

Insights into the survival strategies of these extremotolerant bacteria, thriving under the unique conditions of cleanrooms, were gathered through comprehensive genomic analyses. Genes responsible for the synthesis of compounds such as unknown NAGGN, extensively found in the novel strains, aided the bacteria in facing osmotic stress. The synthesis of NAGGN was induced to enhance bacterial colonization in various ecological niches [48]. This functional property, along with other traits like the presence of genes encoding proteins involved in stress response and adaptation, such as heat shock proteins, cold shock proteins, and chaperones, facilitated survival under harsh cleanroom conditions. This is of particular interest for future NASA missions, where understanding microbial resilience is crucial [49, 50].

To further explore the metabolic potential of the novel species, genes associated with key dissimilatory pathways, particularly those involved in sulfur and nitrogen reduction and dissimilation, were analyzed. The presence of NirB (NADH-dependent nitrite reductase) and NarK (nitrate/nitrite transporter) were identified in multiple species, suggesting potential for nitrite reduction and nitrate/nitrite transport. Our study revealed key genes involved in dissimilatory nitrate reduction-NrfA (cytochrome c nitrite reductase)—present in *Neobacillus phoenicis* 1P10SD and *Neobacillus canaveralius* 3P2-tot-E-2, which is known to be associated with the dissimilatory nitrate reduction to ammonium (DNRA) pathway [51]. Notably, a higher presence of these genes was detected in *Georgenia phoenicis* (1P01AC, 1P07AB) and *Neobacillus canaveralius* (3P2-tot-E-2), suggesting a more extensive genetic potential for dissimilatory nitrate reduction. These findings indicate that alternative nitrogen-based electron acceptors may be utilized by certain novel species, providing a potential survival advantage in the oligotrophic conditions of cleanrooms. A complete overview of gene presence-absence across the analyzed genomes is provided in Supplementary Table S7.

Biofilms are associated with antibiotic resistance, likely due to their organization, which protects bacteria in the inner layers from antimicrobial agents and promotes horizontal gene transfer of resistance genes [52–54]. BolA (COG0271) noticed in proteobacterial members of this study was shown to be highly expressed in bacteria during the stationary phase and under stress conditions, suggesting its role in biofilm formation [55]. Overexpression of BolA in *E. coli* which promoted biofilm formation, while its absence produced thinner biofilms was reported [56]. Stress conditions such as nutrient depletion or oxidative stress resulted in significantly lower biofilm production in BolA mutants compared to the wild-type strain. *Brevundimonas* species during this study also possessed BolA that was reported to be forming biofilms with higher concentrations of antibiotic-resistant bacteria under disinfection pressure from chlorination and chloramination, increasing antibiotic resistance in tap water [57]. The membrane protein YqgA (COG1811) that was found to affect biofilm formation in *E. coli* [58] was also retrieved in the majority of the spore-formers during this study. In *Y.* *pestis*, biofilm formation increased significantly in cobB and yfiQ (COG3936) mutants, suggesting that they were the key players in biofilm formation. The cell fate regulators YmcA, YlbF, and YaaT (COG1744, COG3679, COG4550) were required for sporulation, competence, and biofilm formation [59]. Multiple transcriptional regulators were involved in complex cell differentiation in actinobacteria, cyanobacteria, and sporulating bacillota [60]. Genetic screens for mutants blocked in biofilm formation revealed that ylbF and ymcA genes played crucial roles, with YlbF and YmcA forming a complex with YaaT. Mutants lacking YaaT also showed impaired biofilm formation, competence, and sporulation [59, 61, 62].

*A. phoenicis*, *M. canaveralium*, and *M.* *jepli* genomes had BGCs related to potential production of ε-poly-L-lysine which is a versatile biopolymer with significant potential across various industries due to its strong antimicrobial activity and biodegradability. Its applications range from food preservation to biomedical and industrial uses, making it a valuable compound in enhancing product safety and longevity [63]. Both *Sphingomonas* species (*n* = 2) possess BGCs related to zeaxanthin, a carotenoid produced by other sphingomonads, which is significant for its strong antioxidant properties, protecting cells from oxidative stress [64]. It plays a crucial role in photoprotection by absorbing blue light and preventing damage from UV radiation. In biotechnology, zeaxanthin is valued for its potential health benefits, including eye health, reducing the risk of age-related macular degeneration, and other chronic diseases.

*P. canaveralius* showed BGCs related to the production of bacillibactin, which is a siderophore produced by certain *Bacillus* species [65]. Siderophores are small, high-affinity iron-chelating compounds that microorganisms synthesize and secrete to sequester iron from the environment, which is vital for their growth and metabolism, especially under iron-limiting conditions. *P.* *jepli* contains BGCs related to producing bacillopaline, which is often used in agriculture as a biocontrol agent and biofertilizer. Bacillopaline’s antimicrobial properties can protect plants from pathogenic microorganisms, thus promoting healthier plant growth. By inhibiting plant pathogens, bacillopaline-producing bacterial strains can reduce the reliance on chemical pesticides, offering a more sustainable and environmentally friendly approach to agriculture [66].

All four strains of *Ps.* *phoenicis* exhibited BGCs related to carotenoids, which are reported to serve as powerful antioxidants and photoprotective agents, protecting cells from oxidative damage and UV radiation. They also enhance bacterial survival by aiding quorum sensing and biofilm formation, with significant applications in pharmaceuticals, cosmetics, and food additives [67]. Similarly, both genomes of *G.* *phoenicis* contain BGCs related to alkylresorcinols, which are bioactive compounds known for their antimicrobial, antifungal, and anticancer properties [68]. They play a role in bacterial defense mechanisms and biofilm formation. Additionally, alkylresorcinols are used in pharmaceuticals for their therapeutic potential and in the food industry as natural preservatives due to their inhibitory effects on spoilage organisms. BGCs related to the potential production of paeninodin were found in both strains of *Pe. phoenicis*. Paeninodin is a cyclic lipopeptide produced by *Paenibacillus* species and exhibits significant antimicrobial properties, particularly against Gram-positive bacteria. This compound is noted for its potential in agricultural biocontrol, offering an environmentally friendly alternative to chemical pesticides. Furthermore, surfactant properties of paeninodin make it valuable in industrial applications, such as in the formulation of biosurfactants for bioremediation processes [69].

Using KMAP analysis, several biotechnological applications were predicted in the novel strains. Notably, genes encoding enzymes like polymerases and cellulases, which are relevant for survival in high temperature and alkalinity conditions, were observed. These extremozymes have significant industrial applications due to their stability and efficiency under extreme conditions, making them valuable for processes such as PCR and bioremediation [70, 71]. Further exploration of these POIs from extremotolerant organisms could enhance current industrial processes by comparing them with the best enzymes available, potentially leading to more efficient and robust biotechnological solutions [72].

Culturing methods may introduce biases, favoring certain microbial types over others [73, 74]. However, WGS of novel cultivated species can contribute to metagenome sequence approaches. While comprehensive, technology development is needed for metagenomic analysis to include rare and low-abundant species or those with highly divergent genomes [75]. Future research should focus on further characterizing the functional properties of these novel species, exploring their applications in various industries, and developing improved contamination control strategies.

## Conclusion

The controlled conditions and stringent decontamination processes in cleanrooms create unique selective pressures that foster the survival of rare, stress-resistant microorganisms, leading to the isolation of novel species with significant biotechnological potential. Unlike transient, abundant microbes introduced by human activity, these persistent species endure extreme stressors such as radiation, desiccation, and nutrient limitation, making them highly relevant for NASA’s planetary protection and microbial risk assessments. Cultivating and sequencing these microbes reveals survival mechanisms that metagenomic approaches alone often overlook, providing critical insights into contamination control, space habitat safety, and biotechnological applications. This research directly informs NASA’s forward contamination mitigation strategies, while also benefiting the medical, pharmaceutical, and industrial sectors by identifying microbes relevant to infection control, biocontainment, and antimicrobial resistance. Understanding how rare microbes thrive in extreme environments has profound implications for biotechnology, astrobiology, and human health, particularly in resource-limited settings such as spacecraft and medical cleanrooms.

## Material and methods

Samples were taken from the KSC-PHSF at three distinct times: first before the Phoenix spacecraft arrival on April 25, 2007 (1P), next during the spacecraft’s assembly and testing before its launch on June 27, 2007 (2P), and finally after the spacecraft had been moved to the launch pad on August 1, 2007 (3P). Sample collection and isolation of bacterial strains (*n* = 215 strains) cultured under different extreme conditions were already published [10]. The details of all 53 isolates belonging to novel species, including their isolation locations and conditions, are provided in Supplementary Table S1. Information on culture conditions of seven plate assays and three molecular analyses were already published [5, 10]. Prior to DNA extraction, the isolates were stored at –80 °C in 20% glycerol stock in the JPL microbial collection, ensuring long-term viability and preservation. Additionally, 26 novel species identified in this study were deposited in two recognized culture collections—DSMZ and MTCC—and their respective accession numbers are included in Table 2.

### Controls

Appropriate experimental controls were implemented throughout the study. Sterile water samples served as negative controls for all culture-based assays, while negative controls were included at each procedural step for molecular analyses. Indigenous DNA from sampling materials was analyzed alongside experimental samples. For example, a sterile BiSKit, pre-moistened and exposed to PHSF air for 3–5 min was used as a field control. These field and negative controls underwent the same DNA extraction protocols as surface samples.

For DNA extraction and PCR amplification, controls included blanks (buffer), a DNA-free template (sterile molecular-grade water), and DNA extraction reagents. Purified DNA from *Bacillus* *pumilus* SAFR-032 served as a positive control. Samples lacking expected PCR products in metagenomic library protocols were spiked with 1 ng of *B.* *pumilus* DNA to assess potential PCR inhibitors. Metagenomic libraries were generated for the blanks, subjected to NGS sequencing, and the resulting sequences were deposited in the NCBI SRA database. Details can be obtained from NCBI Project accessions PRJNA1150505 and PRJNA641079.

Shotgun metagenome sequencing analysis produced minimal reads from the negative controls, while culture-based methods did not yield microbial colonies despite testing various culture conditions. However, all 215 isolates characterized in this study were successfully recovered from one of the seven culture conditions employed [10], but only when experimental samples were analyzed. No microbial colonies were observed in the negative control samples, confirming the absence of contamination and the reliability of the cultivation process.

### DNA extraction and whole-genome sequencing

For WGS, genomic DNA was extracted using the ZymoBIOMICS DNA MagBead kit. The DNA of 215 strains (Supplementary Figure S1) was assessed for quality, normalized to 50 ng for library preparation, and barcoded with an Oxford Nanopore Technology transposase barcoding kit (SQK-RBK114.96, Oxford Nanopore, Oxford, UK). Finally, each pool of libraries was loaded onto a PromethION flowcell (FLO-PRO114M, R10.4.1) for long-read sequencing.

### Genome assembly and relatedness indices

Quality checks of the raw reads were conducted using FastQC v.0.12.0 [76], followed by Unicycler v.0.5.0 [77], Flye v.2.9.1 [78], and Canu v.2.2 [79] on the filtered reads for de novo assembly of the genome. To identify the optimal representative assembly from each genome group, genomes within each group were de-replicated using dRep v. 3.4.5 [80]. Subsequently, each assembly was assessed for completeness and contamination by CheckM v.1.2.2 [81].

To facilitate nucleotide-level comparisons of the genomes within their respective genera, the NCBI command line tool datasets v.15.23.0 was employed to obtain all validly described representative genomes of these 18 genera (https://github.com/ncbi/datasets). Then, pairwise ANI was computed using FastANI v.1.34 with the novel strains as a query with representative genomes [82]. Furthermore, for estimating dDDH, the Genome-to-Genome Distance Calculator v.3.0 online tool was used with recommended Formula 2 utilizing the BLAST + alignment tool [83]. In addition, AAI values were computed using aai.rb function from the Enveomics collection toolbox, and the sequence identity for conserved protein *gyr*B was calculated using Blast v.2.13.0, respectively.

### WGS-based phylogeny

For the *Actinomycetota* group (*n* = 11 strains), a set of 138 single-copy genes (SCGs) and *Bacillota* group, 119 SCGs (*n* = 15 strains) were utilized to construct phylogenetic trees at the genus level employing GToTree v.1.8.2 [84]. For *Pseudomonadota* group (*n* = 27 strains), a class level phylogenetic tree was generated using 117 SCGs for *Alphaproteobacteria*, 172 SCGs belonging to *Gammaproteobacteria*, and 203 SCGs of *Betaproteobacteria*. An appropriate outgroup was selected for each tree construction.

Subsequently, IQTREE v.2.2.0.3 [85] was employed with ModelFinder-Plus [86] to construct the phylogenetic tree from the protein alignment generated by GToTree with 1,000 ultrafast bootstrap replicates. Additionally, aiming to place the novel strains in the bacterial tree of life, 4441 complete, non-anomalous representative genomes of bacteria were retrieved from the NCBI Reference Sequence (RefSeq) database. Subsequently, a phylogenetic tree using the 16 SCG-set as previously described by Hug et al. [87] was constructed. All trees were then annotated and visualized using the interactive Tree Of Life (iTOL) v.6.7 [88].

### Microscopic and phenotypic characterizations

Each bacterial strain was cultured on TSA medium incubated at 26 °C for up to 48 h before proceeding for Gram staining [89]. For SEM imaging analysis, the bacterial samples were loaded on silicon wafers and fixed in 4% glutaraldehyde in 0.1 M phosphate buffer for 2 h at room temperature, followed by 3 washes of 5 min with 0.1 M phosphate buffer. The samples were then dehydrated in ascending isopropanol (IPA) and water series (25%, 30%, 50%, 70%, 80%, 90%, 95%, and 100%) each for 10 min, followed by the final 3 times rinsing in 100% IPA and then were critically point dried in EM CPD300 (Leica Company, Wetzlar, Germany). Finally, the silicon wafers carrying the bacterial samples were mounted on SEM stubs (Ted Pella Inc.) using carbon tape and coated with 2 nm of iridium using a sputter coater (Q300T T Plus; Electron Microscopy Sciences Company, Hartfield, PA, USA). The SEM images were collected on Quattro ESEM (ThermoFisher Company, Waltham, MA, USA).

The BioLog GenIII system was used as established by Wragg et al. [15] to generate phenotypic characteristics and MALDI profiles were generated according to the Bielen et al. protocol [16].

### Estimating the abundance of novel species in the cleanroom metagenomes

In order to investigate the presence of newly identified species within controlled cleanroom environments of NASA, 164 metagenome samples were analyzed. They were obtained from Mars 2020 mission assembly cleanrooms: 140 samples from the Spacecraft Assembly Facility (SAF) at the Jet Propulsion Laboratory (JPL), California, and 24 samples from the Payload Hazardous Servicing Facility (PHSF) at the Kennedy Space Center (KSC), Florida. Detailed information about the samples can be found in Supplementary Table S3. The samples treated with propidium monoazide (PMA) were considered for this study to capture only viable and intact cells. Initially, the samples were subjected to quality filtering using fastp v.0.22.0 with a phred-score cut-off of 15 and polyG tails trimming with a minimum length of 10 [90] to eliminate low-quality reads. Then, Bowtie2 v.1.2.2 within MetaCompass v.2.0 were utilized to align the filtered reads to newly identified genomes and determine their abundance in the NASA cleanrooms based on mapped reads. Following this, MEGAHIT v.1.0.6 within MetaCompass was used to assemble the mapped reads and generate consensus sequences [90]. The percentage of reads aligned to these novel species was quantified, and assessed the breadth of coverage of the consensus sequences in each sample.

### Genome characterization and screening of secondary-metabolite biosynthetic potential

Open reading frames (ORFs) in the 53 novel strains were identified by using the command-line tool Prokka v.1.14.5, which employs Prodigal for gene annotation based on multiple reference databases [91]. For functional profiling, Python-based tool cogclassifier v.1.0.5 (https://pypi.org/project/cogclassifier/) was utilized to retrieve Clusters of Orthologous Groups (COGs) from the annotated genomes. To detect antibiotic resistance genes and markers, the Resistance Gene Identifier (RGI) v.6.0.3 was used, leveraging the Comprehensive Antibiotic Resistance Database (CARD) v.3.2.6 [92]. Only “Perfect” and “Strict” matches were considered to ensure high confidence in the identified antibiotic-resistance genes. All genomes were also annotated using the KAUST Metagenomic Analyses Platform (KMAP) [93], which captures Proteins of Industrial Interest (POIs) based on a comprehensive dictionary of genes relevant to industries such as bioprocess engineering, medicine, pharmaceuticals, cosmetics, and detergents.

Secondary metabolite biosynthetic gene clusters (BGCs) were identified in each novel genome using antiSMASH v.7.0.0 [94] with a “Relaxed” detection setting, and the identified BGCs were curated for functional annotation using MIBiG v.3.1 [22]. The study focused on one particular BGC, ε-Poly-L-lysine, present in three of the isolates with 100% similarity score. The gene neighborhood across this cluster was visualized using Clinker on the CAGECAT web server (https://cagecat.bioinformatics.nl/tools/clinker), comparing it with the known producers *Epichloe festucae* and *Corynebacterium* *variabile*. Additionally, the protein sequence of ε-Poly-L-lysine synthetase was aligned using the Clustal Omega web server (https://www.ebi.ac.uk/jdispatcher/msa/clustalo) and visualized the conserved regions in different domains using the NCBI Multiple Sequence Alignment Viewer v.1.25.0.

## Supplementary Information

Additional file 1. Supplementary tables.

Additional file 2. Supplementary figures.

## Data Availability

The draft genome sequences of all the 53 novel strains characterized in this study were deposited in NCBI under BioProject PRJNA1048065. The shotgun metagenome reads are available under BioProject PRJNA1150505 and PRJNA641079. The NCBI accession numbers for the 16S rRNA and WGS are given in Table 1, and the genome versions described in this paper are the first versions. The codes used in this study are available at https://github.com/RamanLab/phoenix-novel-species/wiki. The 26 novel species described in this study were deposited in two recognized culture collections—DSMZ and MTCC—and their respective accession numbers are available in Table 2.
