# Correction: Genomic insights into novel extremotolerant bacteria isolated from the NASA Phoenix mission spacecraft assembly cleanrooms

**DOI:** 10.1186/s40168-025-02274-9

**Published:** 2025-10-21

**Authors:** Júnia Schultz, Tahira Jamil, Pratyay Sengupta, Shobhan Karthick Muthamilselvi Sivabalan, Anamika Rawat, Niketan Patel, Srinivasan Krishnamurthy, Intikhab Alam, Nitin K. Singh, Karthik Raman, Alexandre Soares Rosado, Kasthuri Venkateswaran

**Affiliations:** 1 Biological and Environmental Science and Engineering Division, King Abdullah University of Science and Technology, Thuwal, Makkah, 23955 Saudi Arabia; 2 Department of Biotechnology, Bhupat and Jyoti Mehta School of Biosciences, Indian Institute of Technology Madras, Chennai, 600036 India; 3 Center for Integrative Biology and Systems Medicine (IBSE), Indian Institute of Technology Madras, Chennai, 600036 India; 4 Robert Bosch Centre for Data Science and Artificial Intelligence (RBCDSAI), Indian Institute of Technology Madras, Chennai, 600036 India; 5 MicrobialType Culture Collection and Gene Bank (MTCC), Institute of Microbial Technology, Chandigarh, 160036 India; 6 NASA Jet Propulsion Laboratory, California Institute of Technology, Pasadena, CA USA; 7 Department of Data Science and AI, Wadhwani School of Data Science and AI, Indian Institute of Technology Madras, Chennai, Tamil Nadu 600036 India; 8 Bioscience Program, Biological and Environmental Science and Engineering (BESE), Division, King Abdullah University of Science and Technology (KAUST), Thuwal, Makkah, 23955 Saudi Arabia

**Correction: Microbiome 13, 117 (2025)**

**https://doi.org/10.1186/s40168-025–02082−1**

Following publication of the original article [1], the author reported that there are changes needs to be made to Table 2. Below are the specific changes. Red letters are the changes to be made and blue letters have no changes.

**Table 1:**
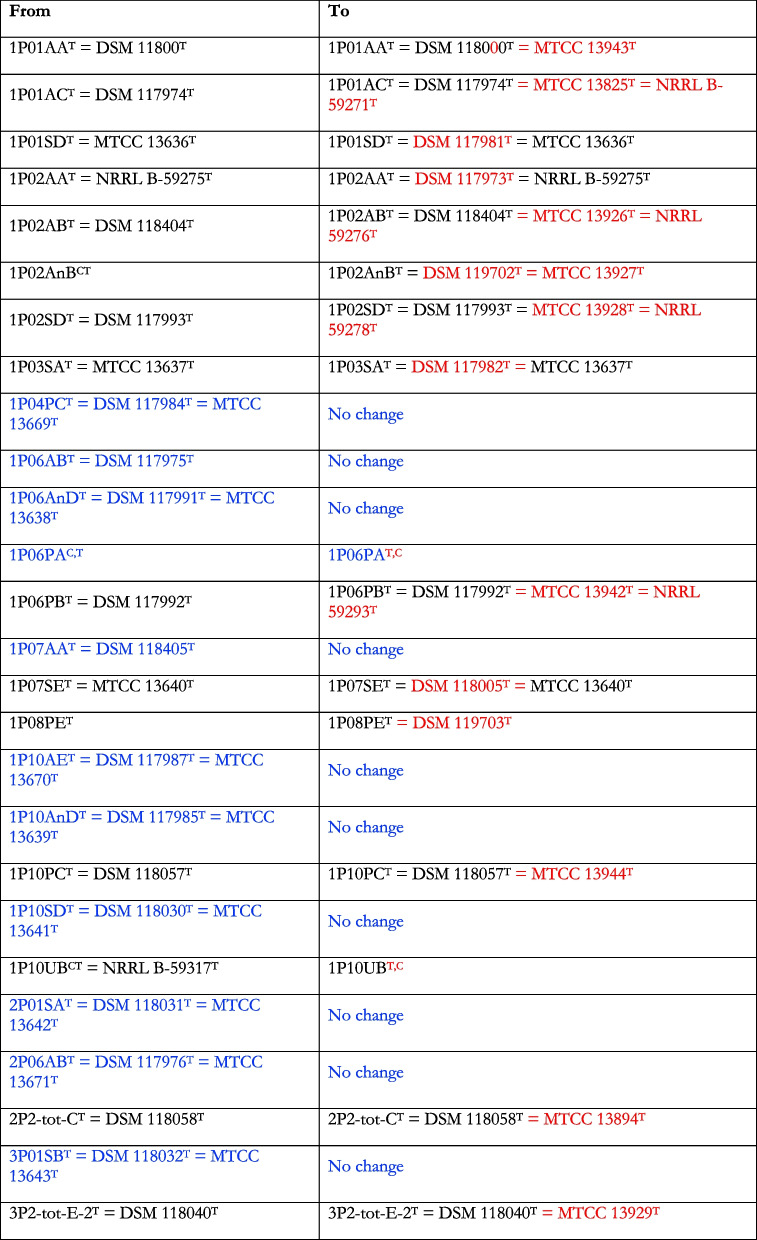


^C^, Cultures were not viable

The original article [1] has been corrected.
