# Supplementary material for: Genomic insights into novel extremotolerant bacteria isolated from the NASA Phoenix mission spacecraft assembly cleanrooms
Source: Microbiome. 2025 May 12;13:117. doi: 10.1186/s40168-025-02082-1 (PMC12067966; doi:10.1186/s40168-025-02082-1)
Supplement: Supplementary file 3 — Additional file 2. Supplementary figures. [file 40168_2025_2082_MOESM2_ESM.pdf]

Supplementary material

Genomic Insights into Novel Extremotolerant Bacteria Isolated from the NASA Phoenix Mission Spacecraft Assembly Facility

Júnia Schultz[1], Tahira Jamil[1], Pratyay Sengupta[2,3,4], Shobhan Karthick Muthamilselvi Sivabalan[4], Anamika Rawat[1], Niketan Patel[1], Srinivasan Krishnamurthy[5], Intikhab Alam[1], Nitin K. Singh[6], Karthik Raman[3,4,7], Alexandre Soares Rosado[1,8*], Kasthuri Venkateswaran[6*]

[1]Biological and Environmental Science and Engineering Division, King Abdullah University of Science and Technology, Thuwal, Makkah, 23955, Saudi Arabia

[2]Department of Biotechnology, Bhupat and Jyoti Mehta School of Biosciences, Indian Institute of Technology Madras, Chennai, 600036, India

[3]Center for Integrative Biology and Systems Medicine (IBSE), Indian Institute of Technology Madras, Chennai, 600036, India

[4]Robert Bosch Centre for Data Science and Artificial Intelligence (RBCDSAI), Indian Institute of Technology Madras, Chennai, 600036, India

[5]Microbial Type Culture Collection and Gene Bank (MTCC), Institute of Microbial Technology, Chandigarh, 160036, India

[6]NASA Jet Propulsion Laboratory, California Institute of Technology, Pasadena, CA, USA

[7]Department of Data Science and AI, Wadhwani School of Data Science and AI, Indian Institute of Technology Madras, Chennai, Tamil Nadu, 600036, India

[8]Bioscience Program, Biological and Environmental Science and Engineering (BESE), Division, King Abdullah University of Science and Technology (KAUST), Thuwal, Makkah, 23955, Saudi Arabia

* These authors share senior authorship.

Correspondence:

kjvenkat1955@gmail.com

alexandre.rosado@kaust.edu.sa

SUPPLEMENTARY FIGURES

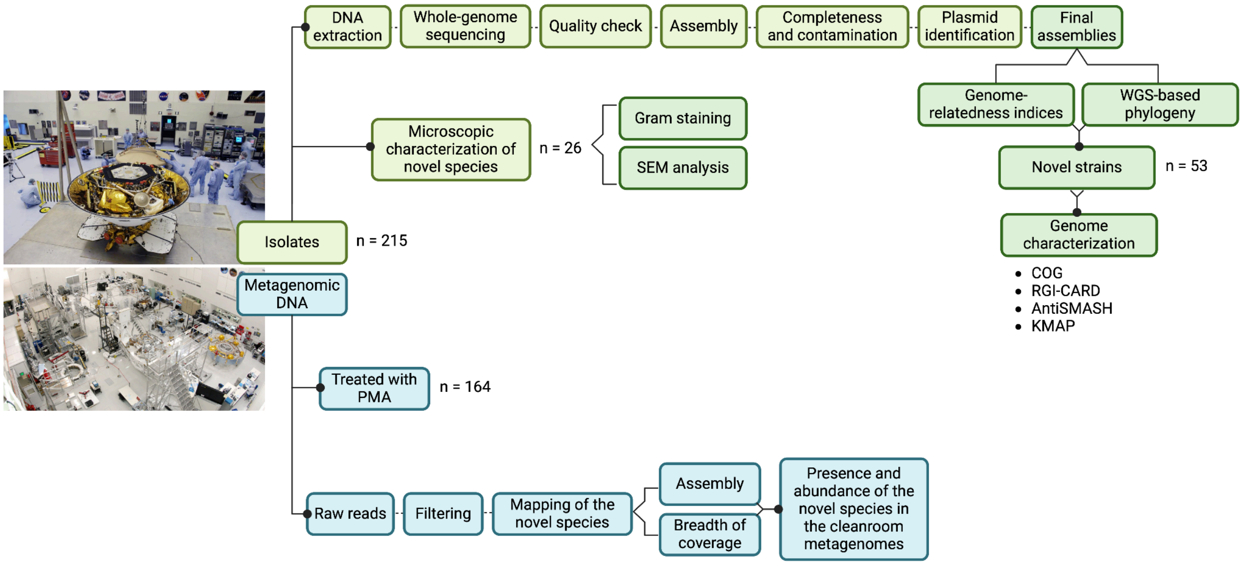

Supplementary Figure S1. A schematic flow chart of the experimental design and analyses used to describe 53 novel strains from the Phoenix mission spacecraft assembly facility, and to evaluate their persistence in the cleanrooms through metagenomic analysis.

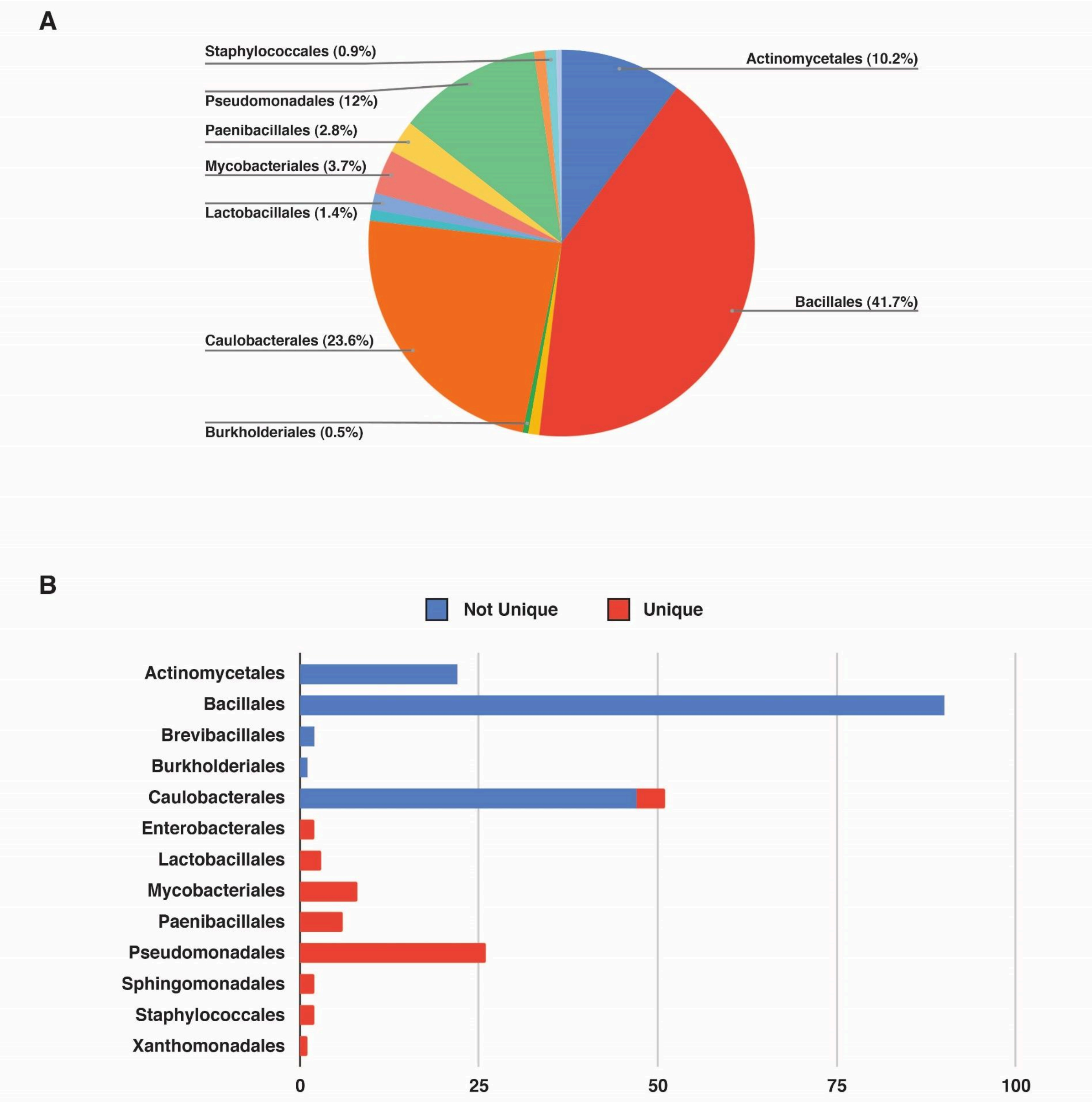

Supplementary Figure S2. Taxonomic distribution of isolated species. A. Order-level classification of 215 isolated species, showing their phylogenetic diversity. B. Proportion of novel species within each order, highlighting the contribution of this study to the discovery of novel species in each taxonomic group.

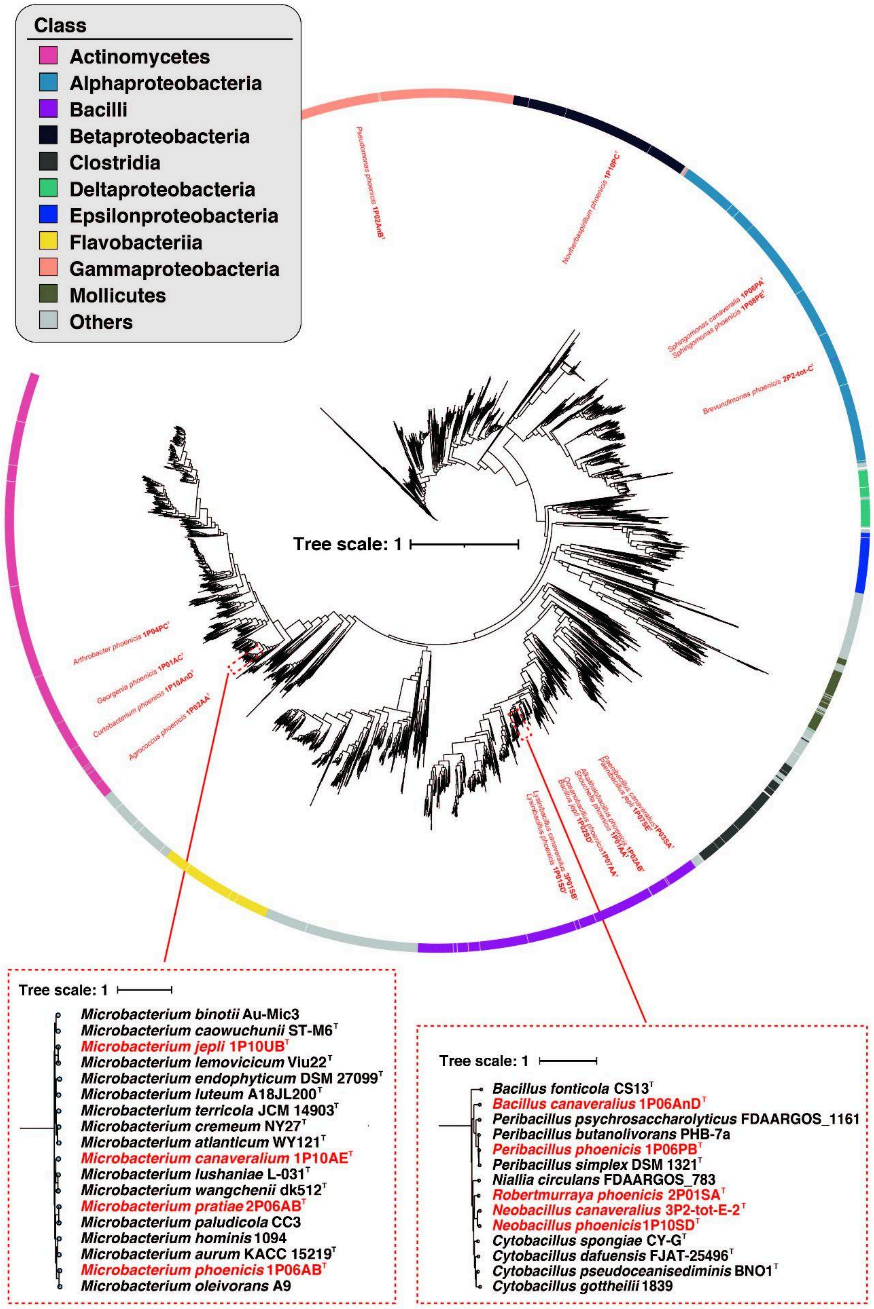

Supplementary Figure S3. A bacterial tree of life containing the 26 novel species isolated from Phoenix mission spacecraft based on 4,441 complete, non-anomalous representative genomes of Bacteria from the NCBI Reference Sequence database and built using the 16 SCG-set as previously described in Hug *et al.* (2016).

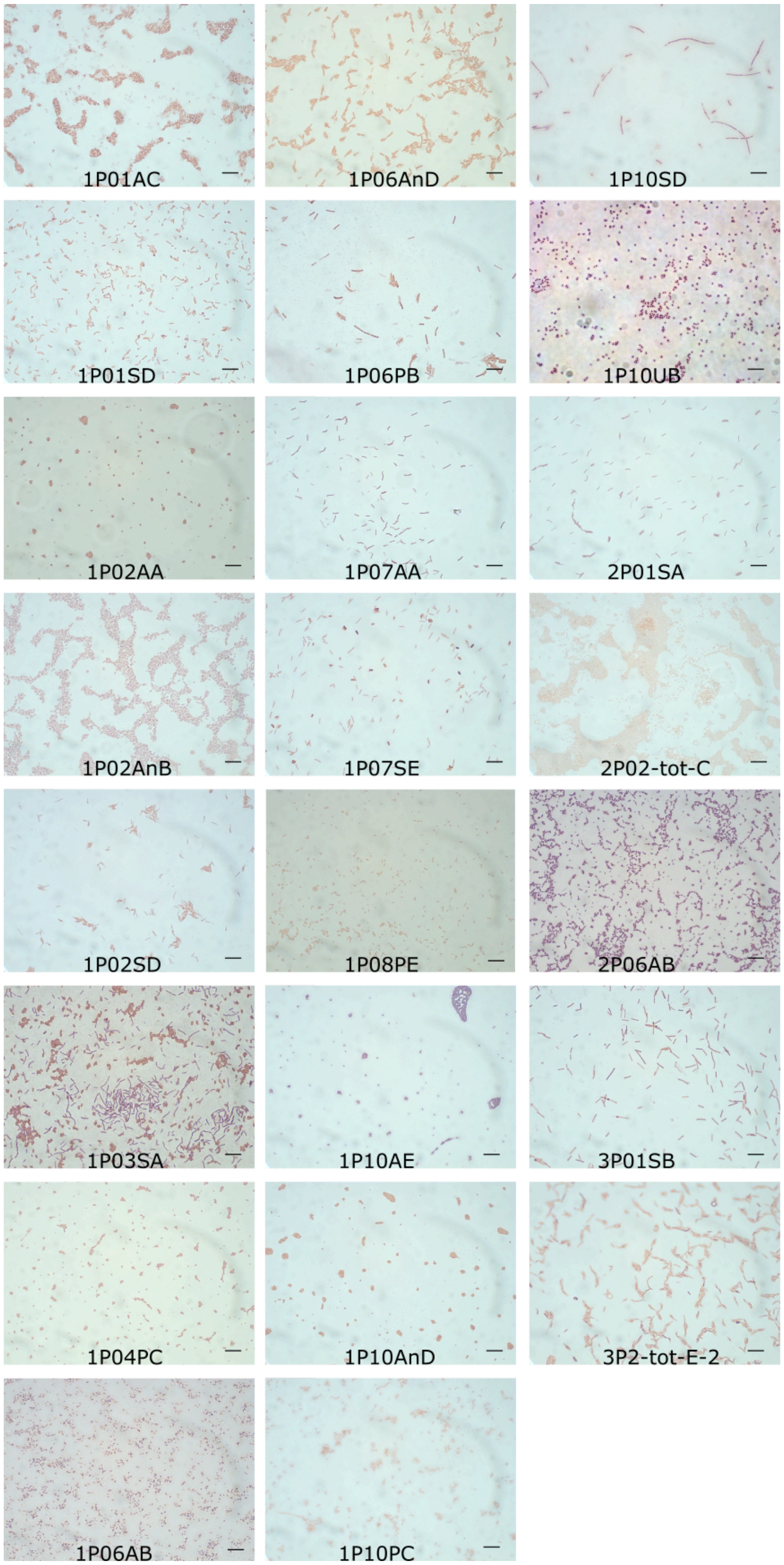

Supplementary Figure S4. Gram-staining images of the novel species isolated from Phoenix spacecraft mission.

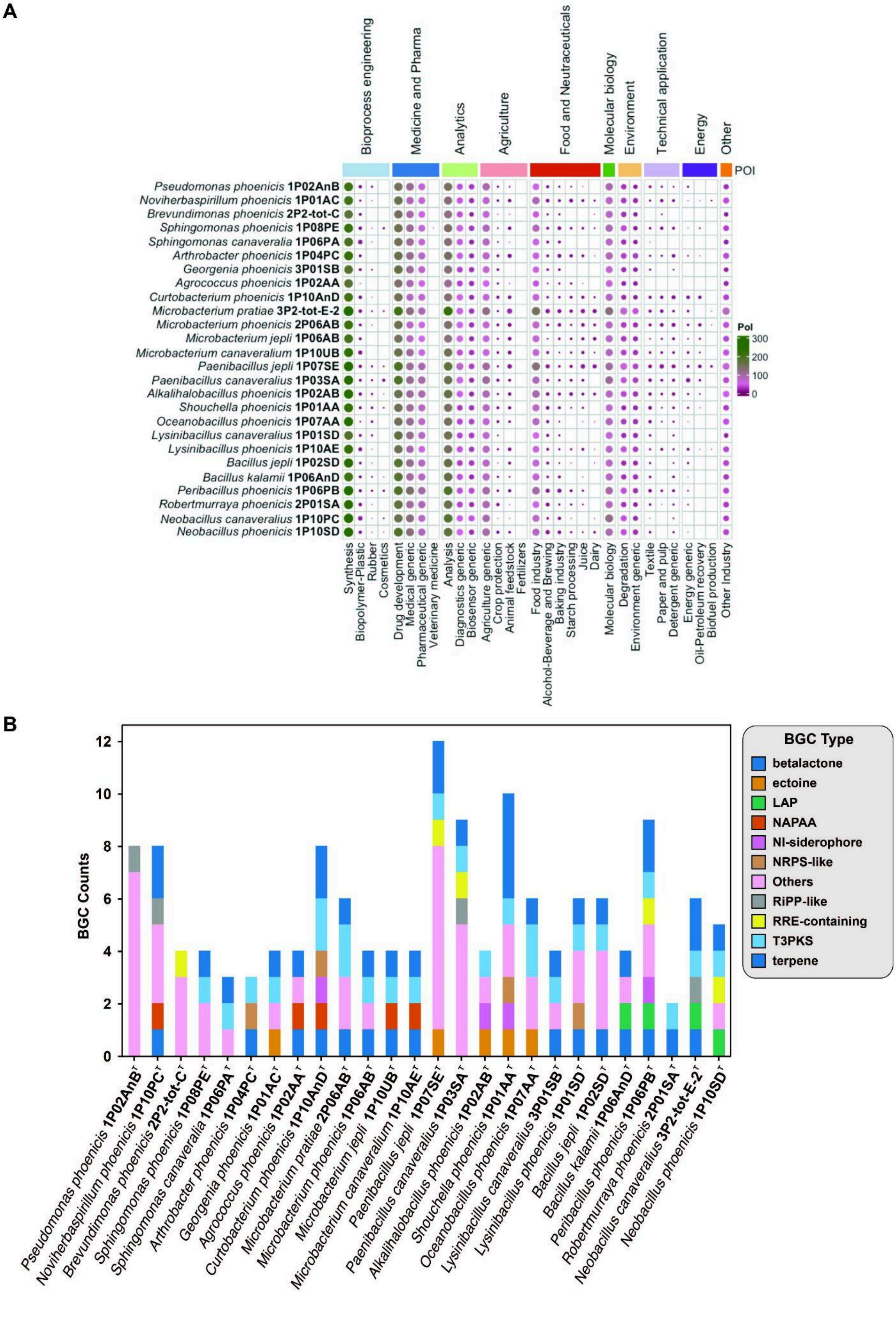
A
Bioprocess engineering
Medicine and Pharma
Analytics
Agriculture
Food and Neutraceuticals
Molecular biology
Environment
Technical application
Energy
Other
POI
Pseudomonas phoenicis 1P02AnB
Noviherbaspirillum phoenicis 1P01AC
Brevundimonas phoenicis 2P2-tot-C
Sphingomonas phoenicis 1P08PE
Sphingomonas canaveralia 1P06PA
Arthrobacter phoenicis 1P04PC
Georgenia phoenicis 3P01SB
Agrococcus phoenicis 1P02AA
Curtobacterium phoenicis 1P10AnD
Microbacterium pratiae 3P2-tot-E-2
Microbacterium phoenicis 2P06AB
Microbacterium jepli 1P06AB
Microbacterium canaveralium 1P10UB
Paenibacillus jepli 1P07SE
Paenibacillus canaveralius 1P03SA
Alkalihalobacillus phoenicis 1P02AB
Shouchella phoenicis 1P01AA
Oceanobacillus phoenicis 1P07AA
Lysinibacillus canaveralius 1P01SD
Lysinibacillus phoenicis 1P10AE
Bacillus jepli 1P02SD
Bacillus kalamii 1P06AnD
Peribacillus phoenicis 1P06PB
Robertmurraya phoenicis 2P01SA
Neobacillus canaveralius 1P10PC
Neobacillus phoenicis 1P10SD
PoI
300
200
100
0
Synthesis
Biopolymer-Plastic
Rubber
Cosmetics
Drug development
Medical generic
Pharmaceutical generic
Veterinary medicine
Analysis
Diagnostics generic
Biosensor generic
Agriculture generic
Crop protection
Animal feedstock
Fertilizers
Food industry
Alcohol-Beverage and Brewing
Baking industry
Starch processing
Juice
Dairy
Molecular biology
Degradation
Environment generic
Textile
Paper and pulp
Detergent generic
Energy generic
Oil-Petroleum recovery
Biofuel production
Other Industry
B
BGC Counts
0
2
4
6
8
10
12
BGC Type
betalactone
ectoine
LAP
NAPAA
NI-siderophore
NRPS-like
Others
RiPP-like
RRE-containing
T3PKS
terpene
Pseudomonas phoenicis 1P02AnB
Noviherbaspirillum phoenicis 1P10PC
Brevundimonas phoenicis 2P2-tot-C
Sphingomonas phoenicis 1P08PE
Sphingomonas canaveralia 1P06PA
Arthrobacter phoenicis 1P04PC
Georgenia phoenicis 1P01AC
Agrococcus phoenicis 1P02AA
Curtobacterium phoenicis 1P10AnD
Microbacterium pratiae 2P06AB
Microbacterium phoenicis 1P06AB
Microbacterium jepli 1P10UB
Microbacterium canaveralium 1P10AE
Paenibacillus jepli 1P07SE
Paenibacillus canaveralius 1P03SA
Alkalihalobacillus phoenicis 1P02AB
Shouchella phoenicis 1P01AA
Oceanobacillus phoenicis 1P07AA
Lysinibacillus canaveralius 3P01SB
Lysinibacillus phoenicis 1P01SD
Bacillus jepli 1P02SD
Bacillus kalamii 1P06AnD
Peribacillus phoenicis 1P06PB
Robertmurraya phoenicis 2P01SA
Neobacillus canaveralius 3P2-tot-E-2
Neobacillus phoenicis 1P10SD

Supplementary Figure S5: A. Identification of Proteins of Interest (PoI) using KMAP with applications in various industries, leveraging the unique functional capabilities of novel species from NASA cleanrooms. B. Secondary metabolite-producing biosynthetic gene clusters of novel Phoenix spacecraft bacterial species identified by AntiSMASH.
